# Candidate Regulatory Relationship and Expression Correlation Between miR-33a-5p and ANK3 in Chronic Myeloid Leukemia

**DOI:** 10.3390/cimb48090949

**Published:** 2026-09-17

**Authors:** Nurgul Kar, Sema Misir, Serap Ozer Yaman, Osman Akidan, Ceylan Hepokur, Yuksel Aliyazicioglu

**Affiliations:** 1 Department of Biochemistry, Faculty of Pharmacy, Sivas Cumhuriyet University, 58140 Sivas, Türkiye; 2 Department of Medical Biochemistry, Trabzon Kanuni Training and Research Hospital, Faculty of Medicine, Trabzon University, 61250 Trabzon, Türkiye; 3 Department of Hematology, Mugla Training and Research Hospital, 48000 Mugla, Türkiye; 4 Department of Medical Biochemistry, Faculty of Medicine, Karadeniz Technical University, 61080 Trabzon, Türkiye

**Keywords:** chronic myeloid leukemia, microRNA, miR-33a, ankyrin 3

## Abstract

Chronic myeloid leukemia (CML) is a type of bone marrow cancer characterized by the uncontrolled proliferation of myeloid cells. MicroRNAs (miRNAs) are small, non-coding RNA molecules that play a crucial role in the post-transcriptional regulation of gene expression. This study aims to examine the association between miR-33a-5p and Ankyrin 3 (ANK3) in CML and to investigate the regulatory mechanisms of miR-33a-5p in the progression of this disease. mRNA expression profiles were obtained from the GSE100026 dataset within the Gene Expression Omnibus (GEO) repository. Quantitative real-time polymerase chain reaction (RT-qPCR) was conducted to assess the expression levels of miR-33a-5p and ANK3. To investigate the regulatory mechanism of miR-33a-5p/ANK3, various databases such as miRNet, miRDIP, TargetScan, BioGRID, and CancerSEA were utilized. The expression of miR-33a-5p was markedly elevated, while ANK3 expression was significantly reduced. Furthermore, pathway clustering and functional assessments of ANK3 demonstrated its involvement in regulating the cell cycle and apoptosis. The findings identify an inverse expression pattern between miR-33a-5p and ANK3 across the two cell models and support a candidate regulatory relationship that warrants direct functional validation. This relationship may merit further investigation as a potential molecular target in CML.

## 1. Introduction

Chronic myeloid leukemia (CML) is a type of leukemia characterized by the u*n*controlled and abnormal proliferation of bone marrow cells at various stages of maturation [1]. It accounts for approximately 15% of all leukemia cases diagnosed in the adult population [2]. A hallmark of CML is the presence of the Philadelphia chromosome (Ph), an aberrant chromosome formed due to a reciprocal translocation between the long arms of chromosomes 9 and 22. This genetic alteration gives rise to the BCR-ABL1 fusion gene [3]. The resulting BCR-ABL1 chimeric protein acts as a persistent tyrosine kinase, activating various signaling pathways that contribute to malignant cell transformation [4]. The development of BCR-ABL1 tyrosine kinase inhibitors (TKIs) has been a significant advancement in the treatment of CML [5]. However, ongoing challenges highlight the increasing importance of patient-specific factors in selecting the most appropriate therapy [6]. Therefore, a comprehensive understanding of the underlying molecular mechanism of CML and a commitment to developing targeted therapies remain crucial [3]. Moreover, early disease detection is critical in enhancing overall survival outcomes for patients [7].

MicroRNAs (miRNAs) are small, non-coding RNA molecules, approximately 22 nucleotides in length, that are evolutionarily conserved and play essential roles in gene regulation. Aberrant expression of miRNAs has been implicated in the onset and progression of various cancer types [8]. miRNAs primarily regulate gene expression by directly binding to target mRNAs’ 3′-untranslated regions (3′-UTRs), leading to mRNA degradation and/or translational inhibition [9]. Through these mechanisms, miRNAs i*n*fluence cancer cell growth, proliferation, migration, and invasion [10,11]. Identifying miRNAs and their target genes in CML is crucial for understanding their roles in tumor initiation and progression [12]. Recent studies suggest that miRNAs could serve as potential therapeutic targets in the development and treatment of CML [11,13]. As a member of the evolutionarily conserved miR-33 family, miR-33a is an intronic miRNA embedded within sterol regulatory element-binding protein genes. Aberrant expression levels of miR-33a have been linked to multiple diseases, including cancer. miR-33a has been identified as a key regulator in various cancer types, potentially acting as either a tumor suppressor or an oncogene, depending on its target genes. Studies indicate that miR-33a and its target genes influence several biological processes in tumor cells, such as proliferation, metastasis, migration, invasion, cell cycle regulation, apoptosis, self-renewal, chemotherapy resistance, and radioresistance. Moreover, miR-33a has been associated with patient outcomes, including prognosis and survival [14]. However, current data on the expression, function, and target genes of miR-33a-5p in CML remain limited.

Ankyrin 3 (ANK3), also known as ankyrin-G, is a member of the ankyrin protein family [15]. The ANK3 protein functions as a bridge between the plasma membrane and the cytoskeleton by linking spectrin proteins to integral membrane proteins. It regulates various cellular processes, including cell motility and proliferation [16]. Genetic and expression alterations of ANK3 have been reported in various cancer types. The downregulation of ANK3 has been associated with poor prognosis in breast, prostate, lung, and ovarian cancers [15,16]. In prostate cancer cells, ANK3 has been shown to regulate the cell cycle and inhibit cell invasion [16], while its overexpression has been found to induce apoptosis and suppress epithelial–mesenchymal transition in papillary thyroid carcinoma cells [17]. These findings highlight the tumor-suppressive functions and prognostic significance of ANK3 in various malignancies. However, its role in CML remains largely undefined. Consequently, further in-depth studies are necessary to investigate the functional mechanisms and molecular targets of ANK3 in CML.

Bioinformatic techniques are utilized to identify diseases’ pathophysiology and determine pharmacological treatment targets at the genetic and protein levels [18]. In cancer studies, tumor bioinformatics holds a pivotal position. The systematic integration and analysis of genomic, transcriptomic, epigenomic, and other omics datasets contribute to a deeper comprehension of cancer biology. These resources facilitate the exploration of molecular mechanisms, personalized treatment approaches, and cancer prognosis [19].

This study aimed to investigate the expression alterations in miR-33a-5p and ANK3 in CML through the integration of multiple data sources and the application of in vitro methods. Furthermore, we sought to explore the potential relationship between miR-33a-5p and ANK3 and its possible relevance to CML progression.

## 2. Materials and Methods

### 2.1. Identification of miRNAs in CML

For the detection of miRNAs in CML, miRNet (https://www.mirnet.ca/, accessed on 8 January 2025), an online platform, was used. CML BCR-ABL positive was selected from the miRNet disease tab [20].

### 2.2. Prediction of miRNA Targets

We used TargetScan (https://www.targetscan.org/vert_80/, accessed on 9 January 2025) [21] and mirDIP (https://ophid.utoronto.ca/mirDIP/, accessed on 9 January 2025) [22] to ide*n*tify potential target genes of hsa-miR-33a-5p. In the mirDIP database, we selected the “very high” score class for determining miR-33a-5p targets. Additionally, using TargetScan, we visualized the complementary sequences between ANK3 3′UTR and miR-33a-5p [21]. To construct a visual network of miRNA–target interactions, we employed Cytoscape software (version 3.10.3).

### 2.3. Differentially Expressed Genes (DEGs) in CML and Data Processing

The dataset used in this study was obtained from the National Center for Biotechnology Information (NCBI) Gene Expression Omnibus (GEO) (http://www.ncbi.nlm.nih.gov/geo, accessed on 9 January 2025) [23].

The data from GSE100026 included mRNA profile changes from ten CML patient samples (comprising 5 chronic-phase [CP] and 5 blast-phase [BP] patients) and five normal peripheral blood samples, all of which were analyzed using high-throughput RNA sequencing (RNA-seq). This analysis aimed to identify differentially expressed and spliced transcripts associated with chronic myeloid leukemia [24]. GEO2R (https://www.ncbi.nlm.nih.gov/geo/geo2r/, accessed on 9 January 2025) was used to identify differentially expressed mRNAs between CML and the control group [25]. GEO2R applies multiple-testing correction to control the false-positive rate. Differentially expressed mRNAs were selected using an adjusted *p*-value < 0.05 and |logFC| > 4.

### 2.4. Single-Cell Analysis

CancerSEA (http://biocc.hrbmu.edu.cn/CancerSEA/home.jsp, accessed on 9 January 2025) provides insights into the distinct functional states of cancer cells at the single-cell level [26]. The relationships between ANK3 expression and various functional states of cancer cells at the single-cell level were examined using CancerSEA.

### 2.5. Interaction Network Analysis of ANK3 and Functional Enrichment Analysis

The protein–protein interaction (PPI) network was retrieved from BioGRID (Biological General Repository for Interaction Datasets (https://thebiogrid.org/, accessed on 13 January 2025) [27]. The Database for Annotation, Visualization, and Integrated Discovery (DAVID; http://david.ncifcrf.gov/, accessed on 13 January 2025) was employed to conduct pathway and functional analyses of the potential activities of the selected genes (ANK3, CTNNB1, KRAS, CCNF, and TP53) [28]. Gene Ontology (GO), Kyoto Encyclopedia of Genes and Genomes (KEGG), and Reactome databases were used to identify biological processes and pathway enrichment. Enrichment terms were identified using DAVID software, and a raw *p*-value threshold of *p* < 0.05 was strictly applied as the cutoff for statistical significance in all reported tables.

### 2.6. Cell Culture

Human chronic myelogenous leukemia (CML) (K-562) (ATCC, CCL-243TM) from Dr. Gulden Yorgancioglu Budak (Istinye University) and human keratinocyte cells (HaCaT) were obtained from Atlas Biotechnology. All cells were cultured in Dulbecco’s Modified Eagle Medium (DMEM) supplemented with 10% fetal bovine serum (FBS), penicillin (192 U/mL), and streptomycin (200 µg/mL) under standard conditions of 5% CO_2_ and 37 °C with maintained relative humidity. Once the cells reached approximately 80% confluence, they were harvested for further analysis and experimentation.

### 2.7. RNA Extraction and Quantitative Real-Time PCR (RT-qPCR) for ANK3

Total RNA was extracted from all collected cells using the RNA Isolation Kit (GeneAll, RiboEx, Cat: 301-001, Seoul, Korea) according to the manufacturer’s instructions. Extracted total RNA purity and concentration were assessed spectrophotometrically (A260/A280 ratio between 1.8 and 2.0) using a microvolume spectrophotometer (Thermo NanoDrop 2000, Wilmington, DE, USA). cDNA synthesis was performed using a cDNA Synthesis Kit with RNase Inhibitor (High Capacity) (A.B.T., Cat: C03-01-05, Ankara, Turkey), following the manufacturer’s protocol. The reaction mixtures were incubated at 25 °C for 10 min, 37 °C for 120 min, and 85 °C for 5 min. RT-qPCR was conducted using A.B.T. 2X SYBR Green Mastermix (A.B.T., Cat: Q03-01-05, Ankara, Turkey) in accordance with the manufacturer’s instructions. Each PCR (20 µL) contained 4 µL of cDNA, 1 µL (10 μM) of forward primer, 1 µL (10 μM) of reverse primer, 1 µL of ROX, 3 µL of sterile water, and 10 µL (2X) SYBR master mix. The following primers were used: ANK3-F 5′-ACACCTTGAACAGAAGCTCCTA-3′, ANK3-R 5′-CGTCCACCATAAAGCTAACCAG-3′, GAPDH-F 5′-TGA CTT CAA CAG CGA CAC CCA-3′, and GAPDH-R 5′-CAC CCT GTT GCT GTA GCC AAA-3′ (Oligomer Biotechnology, Çankaya, Ankara, Turkey). The expression levels of ANK3 were normalized to the amount of GAPDH in the same sample. The reaction mixtures were initially incubated at 95 °C for 5 min, followed by 40 cycles of 95 °C for 15 s and 60 °C for 60 s. Ct (threshold cycle) values were determined using the 2^−ΔΔCt^ method [29].

### 2.8. miRNA Extraction and RT-qPCR Analysis

The expression of miR-33a-5p was evaluated using real-time polymerase chain reaction (PCR) according to the manufacturer’s instructions. miRNA fractions and purities were measured using a micro volume spectrophotometer (Thermo NanoDrop 2000, Wilmington, DE, USA). cDNA was synthesized following the manufacturer’s protocol using the High-Capacity cDNA Synthesis Kit (A.B.T., Cat: C03-01-05, Ankara, Turkey). For target-specific pre-amplification, diluted cDNA (1:4) was used with the miScript primer (miR-33a-5p) and the miScript PreAMP PCR Kit (Qiagen, Cat: 331452, Hilden, Germany). U6 snRNA was used as the internal control. A 20 µL reaction mixture was prepared by combining 5 µL of cDNA template with SYBR Green Master Mix (Qiagen, Cat: 218073, Hilden, Germany) and miScript primer assays (Qiagen, Cat: 218300, Hilden, Germany). This mixture was added to a custom 96-well miScript miRNA PCR plate containing forward and reverse primers specific to miR-33a-5p. The real-time PCRs were performed using the LightCycler 480 system (Roche, Basel, Switzerland). The PCR co*n*ditions were as follows: initial denaturation at 95 °C for 15 min, followed by 40 cycles of 94 °C for 15 s, 55 °C for 30 s, and 70 °C for 34 s. This was followed by a final step at 95 °C for 1 s, 60 °C for 1 min, and a cooling cycle at 95 °C. The cycle threshold (CT) values, representing the number of cycles required for the fluorescence signal to exceed the background noise threshold, were determined. ΔCt values were inversely related to miRNA expression levels. The relative expression levels of miRNA were calculated using the comparative threshold cycle 2^−ΔΔCt^ method, with normalization to the internal reference U6 snRNA (U6 snRNA-F: 5′-CTCGCTTCGGCAGCACA-3′, U6 snRNA-R: 5′-AACGCTTCACGAATTTGCGT-3′) [29].

### 2.9. Statistical Analysis

Statistical analyses were conducted using IBM SPSS Statistics for Windows (version 23.0; IBM Corp., Armonk, NY, USA). The normality of the variables was assessed using the Kolmogorov–Smirnov test. Expression levels of miR-33a-5p and ANK3 were compared between K562 and HaCaT cells using the independent sample *t*-test. Because the expression data used for association analysis were non-normally distributed, Spearman rank correlation was used. The exploratory correlation analysis pooled the K562 and HaCaT observations (*n* = 18) to describe their overall expression pattern across the two cell models; it was not interpreted as a within-cell line co-regulatory relationship. Statistical significance was set at *p* < 0.05.

## 3. Results

**Figure 1:**
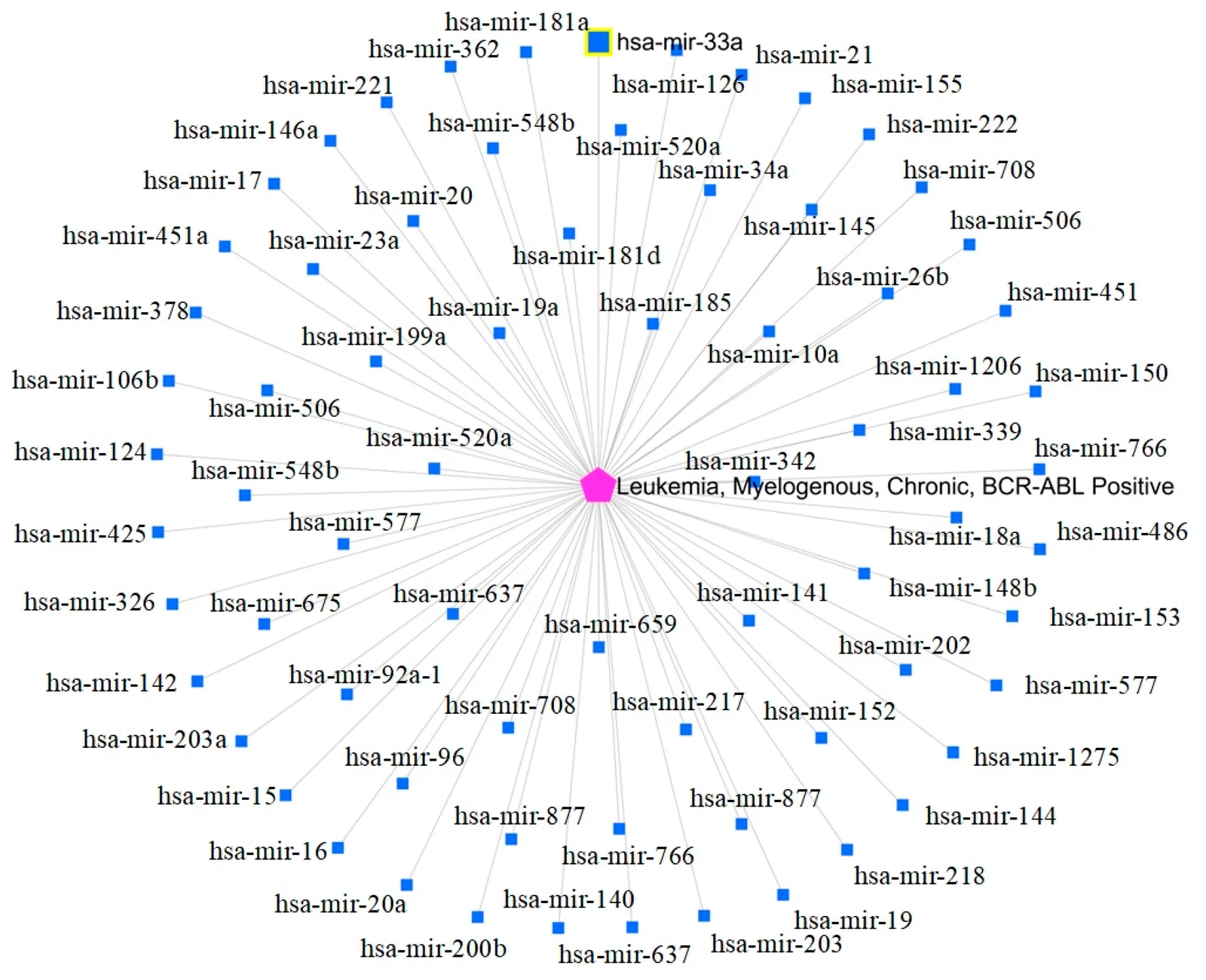
CML-associated miRNAs identified using miRNet. The central pink node represents CML, and the blue nodes represent associated miRNAs.

**Figure 2:**
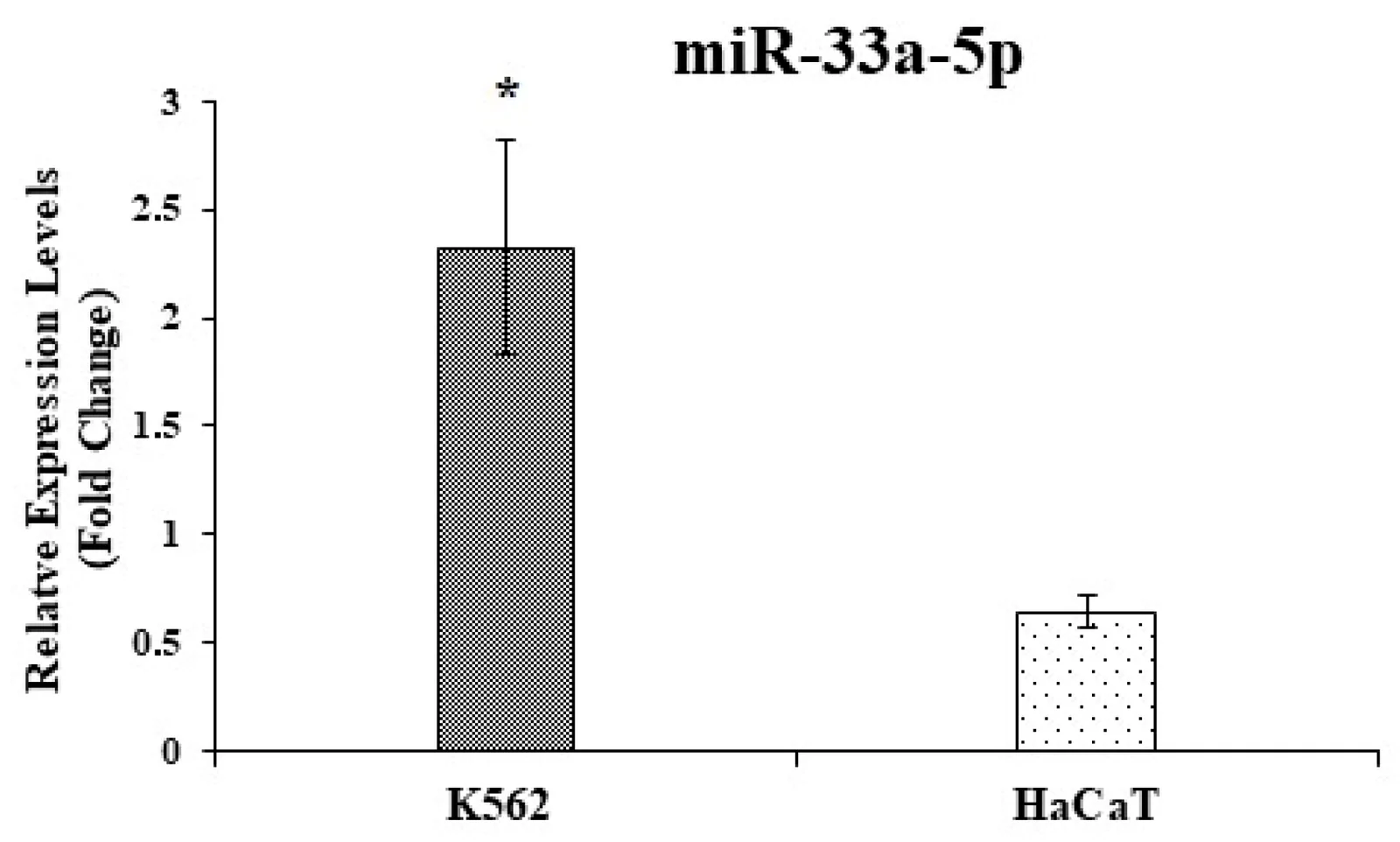
Relative miR-33a-5p levels in K562 and HaCaT cells. RNU6 was used for the normalization of miRNAs. * Represents statistically significant difference (*p* < 0.0001) compared to HaCaT control cells (independent-samples *t*-test, *n* = 9).

The miRNet database was utilized to identify CML-associated miRNAs (Figure 1). Seventy-one miRNAs were identified, and miR-33a was selected for further analysis. The expression levels of miR-33a-5p were evaluated in K562 and HaCaT cells using RT-qPCR. The results revealed a significantly higher expression of miR-33a-5p in K562 cells compared to HaCaT cells (Figure 2).

**Figure 3:**
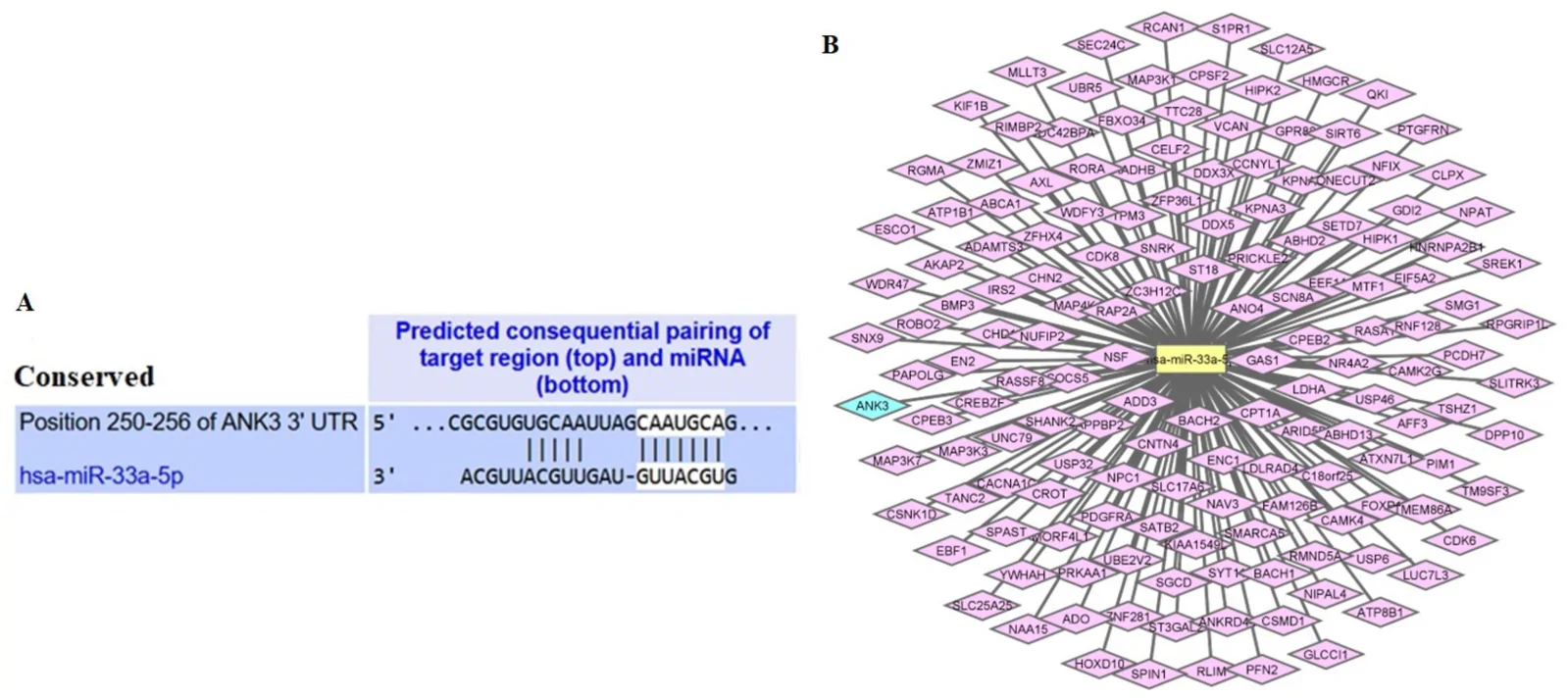
Targeting relationship between miR-33a-5p and ANK3. (**A**) The complementary sequences of ANK3 3′UTR and miR-33a-5p were presented by TargetScan. (**B**) The miR-33a-5p target visualization network contains 153 identified common targets.

Common targets of miR-33a-5p were identified using two different miRNA target prediction tools (TargetScan, mirDIP), and common targets were visualized with Cytoscape software. According to the results of these databases, 523 genes were identified in TargetScan, 194 in mirDIP, and 153 as common target genes of miR-33a-5p (Table S1 and Table S2). One hundred fifty-three common genes were visualized with Cytoscape (Figure 3). The ANK3 gene, whose expression is decreased in CML, was selected for further analysis among these target genes.

**Figure 4:**
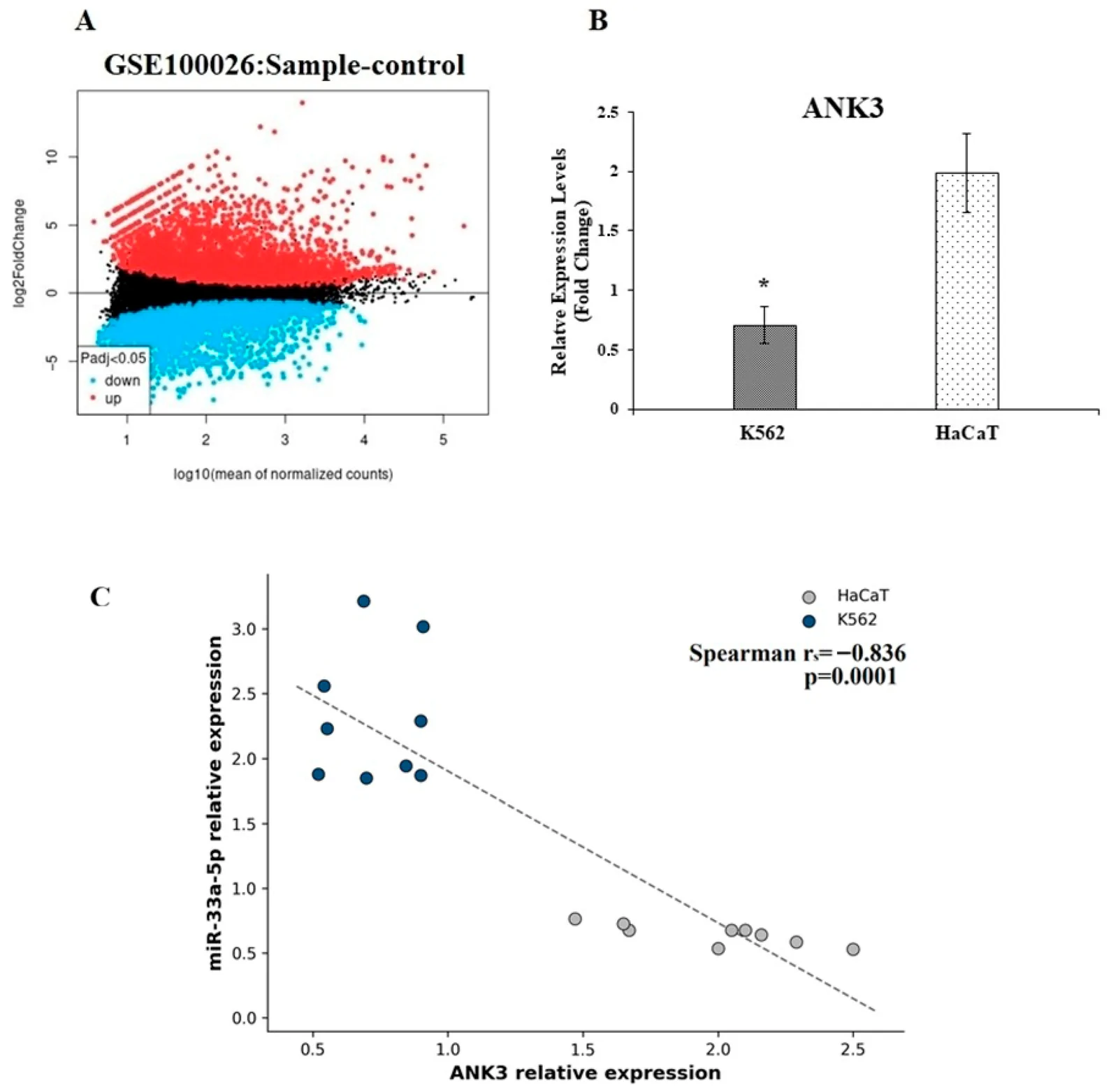
(**A**) Volcano plot showing genes differentially expressed in CML compared with normal controls. Blue dots indicate downregulated mRNAs, and red dots indicate upregulated mRNAs. (**B**) ANK3 gene expression assessed by RT-qPCR in K562 and HaCaT cells. GAPDH was used for normalization. * Indicates a statistically significant difference compared with HaCaT control cells (*p* < 0.0001; independent sample *t*-test; N = 8 per group). (**C**) Exploratory pooled Spearman correlation between miR-33a-5p and ANK3 expression across K562 and HaCaT observations (*n* = 18, r_s_ = −0.836, *p < 0.0001). Colors identify* the two cell lines. The pooled coefficient should not be interpreted as a within-cell line association.

To establish a scientifically rigorous and biologically relevant downstream target for miR-33a-5p in CML, we implemented a systematic dual-filtering pipeline intersecting in silico predictions with clinical transcriptome data. First, we performed target prediction analysis using TargetScan and mirDIP, which identified 523 and 194 potential target genes, respectively, yielding 153 overlapping candidate genes targeted by hsa-miR-33a-5p. Concurrently, to identify highly dysregulated genes in clinical CML, we analyzed the GSE100026 dataset. Applying stringent statistical thresholds of an adjusted *p*-value < 0.05 and |logFC| > 4, we isolated 84 upregulated and 112 downregulated genes in CML samples compared to normal controls (Figure 4A). By intersecting the 153 predicted target genes with the 112 clinically downregulated genes, ANK3 emerged as the primary candidate of interest. ANK3 exhibited a profound decrease in expression with a fold change of −4.31, supporting its prioritization as a candidate for further in vitro investigation. Moreover, analysis of ANK3 gene expression in K562 and HaCaT cells revealed significantly lower transcript levels in K562 cells (*p* = 0.0001; Figure 4B). An exploratory Spearman analysis pooling the K562 and HaCaT observations showed a significant inverse association between miR-33a-5p and ANK3 expression (*n* = 18, r_s_ = −0.836, *p* < 0.0001; Figure 4C). This association reflects the overall separation of the two cell models and was not interpreted as a within-K562 co-regulatory relationship.

**Figure 5:**
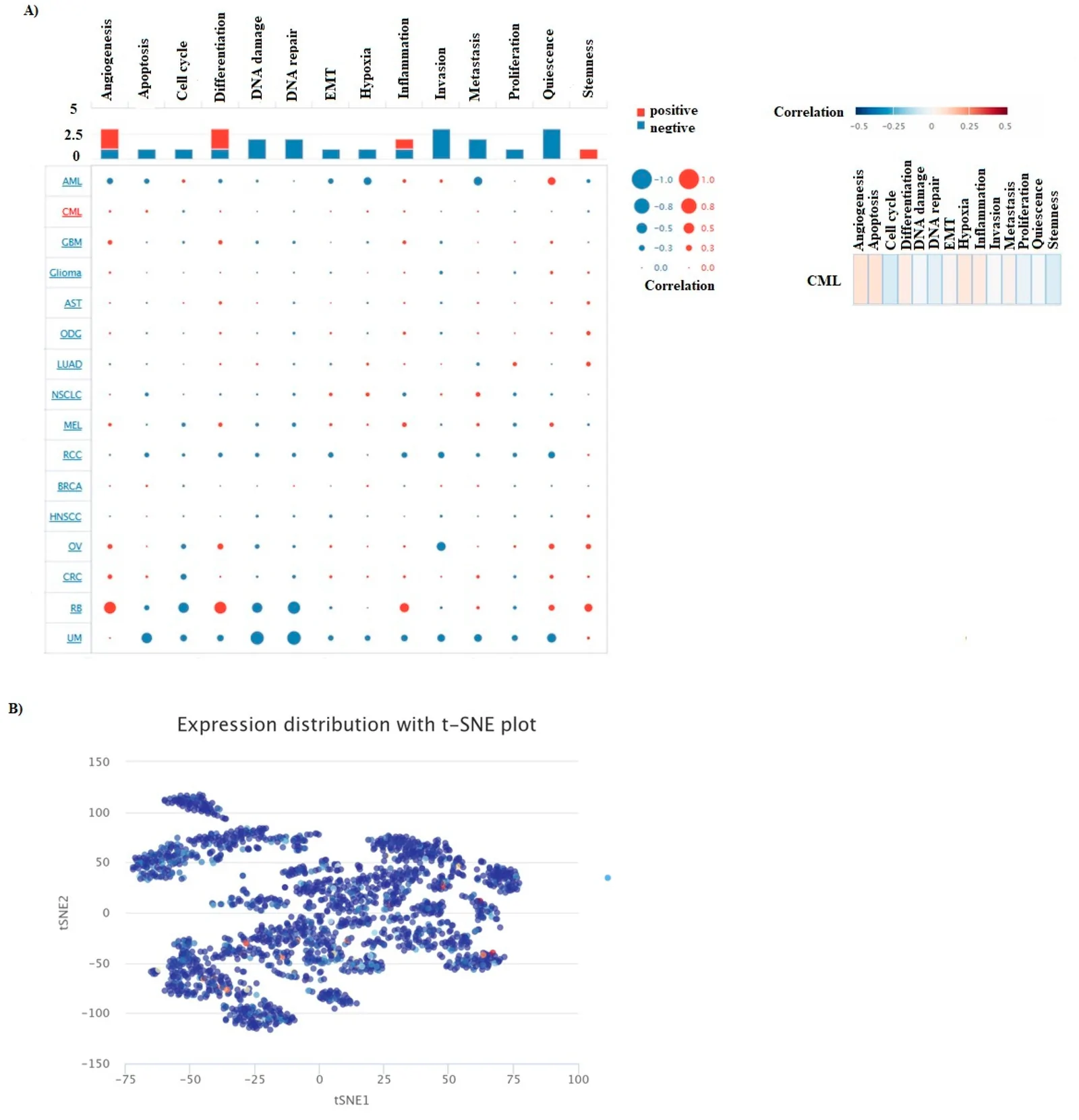
Correlation of ANK3 expression with functional states in CancerSEA datasets. (**A**) Bubble chart showing correlations between ANK3 expression and functional states across cancer datasets, including CML. (**B**) Single-cell ANK3 expression profile in CML visualized using t-SNE.

We analyzed the relationship between ANK3 and the functional states of CML cancer using single-cell line data from CancerSEA (Figure 5). The function of ANK3 in CML was investigated in 14 different states, including angiogenesis, apoptosis, invasion, EMT, differentiation, proliferation, DNA damage, metastasis, hypoxia, inflammation, cell cycle, DNA repair, stem cell, and quiescence. For CML, these values are 0.075, 0.076, −0.071, 0.043, −0.007, −0.039, 0.015, 0.058, 0.048, −0.002, 0.024, −0.03, 0, and −0.062, respectively. In addition, the T-SNE diagram was used to display the expression profile of ANK3 in CML.

**Figure 6:**
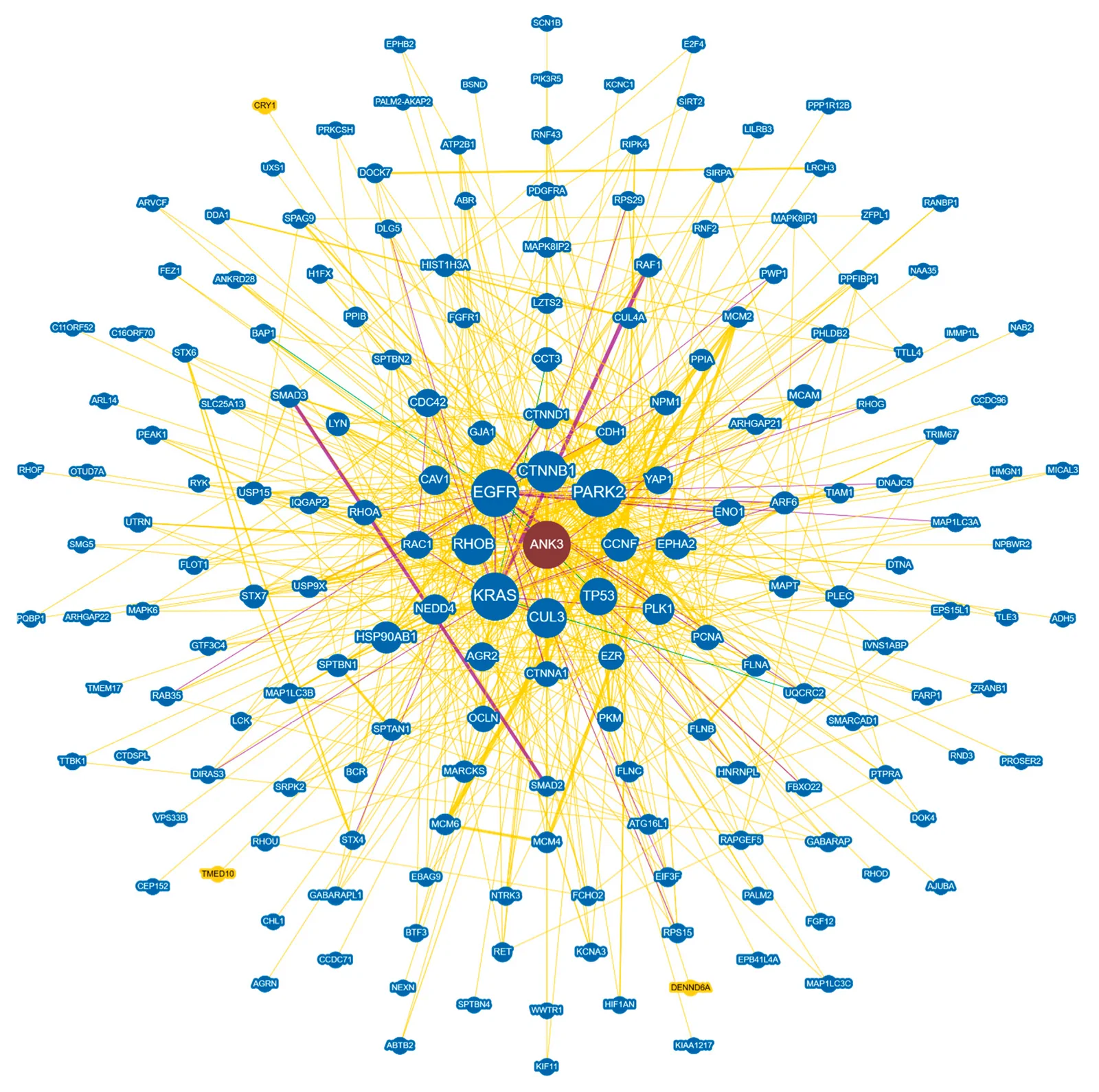
Protein interaction network based on ANK3 from BioGRID (green: association with genetic evidence, yellow: association with physical evidence, purple: association with genetic and physical evidence).

PPI analysis can reflect the molecular mechanisms underlying physiological and pathological changes in cancer. The PPI network of Ank3-associated target genes was obtained from the BioGRID database (Figure 6). Subsequently, five genes with high node degrees associated with cancer pathology (ANK3, CTNNB1, KRAS, CCNF, and TP53) were selected as central genes for further analysis. GO and KEGG pathway analyses were performed to understand better the pathways and processes influenced by the identified genes.

For Biological Processes (BPs), these genes were mainly enriched in processes satisfying the statistical cutoff (*p* < 0.05), such as positive regulation of gene expression, neuroblast proliferation, stem cell proliferation, Ras protein signal transduction, neuron apoptotic process, MAPK cascade, and regulation of the cell cycle, among others. For Molecular Functions (MFs), the results indicated that these genes were primarily i*n*volved in processes including disordered domain-specific binding, transmembrane transporter binding, and RNA polymerase II-specific DNA-binding transcription factor binding (Table 1). KEGG and Reactome pathway analyses revealed that these genes were significantly enriched (*p* < 0.05) in pathways related to proteoglycans in cancer, pathways in cancer, chronic myeloid leukemia, apoptosis, and the thyroid hormone signaling pathway, as well as developmental biology, Ca^2+^ pathway, and beta-catenin i*n*dependent WNT signaling, among others (Table 2).

## 4. Discussion

Chronic myeloid leukemia is one of the most common types of leukemia observed in the adult population [30]. Although significant progress has been made in understanding the molecular mechanisms underlying CML development [31], the prognosis for patients remains unfavorable [32]. While TKIs have made it possible to manage key treatment objectives—such as prolonging survival, improving quality of life, and preventing progression to the blast phase—several challenges persist [2]. Among these unresolved issues are the necessity for most patients to continue TKI therapy indefinitely to prevent relapse, chronic TKI-related toxicities, and the impact of TKIs on overall quality of life [30]. Addressing these challenges requires a deeper understanding of the molecular pathways involved in CML pathogenesis. Numerous studies have demonstrated that CML arises from the interplay of multiple genes, pathways, and contributing factors [33]. Studies have indicated that miRNAs can exhibit oncogenic or tumor-suppressive pro*p*erties in different types of cancer [34]. The expression of miRNAs is a dynamic process that reflects cellular physiological changes [35]. Recent studies have demonstrated a strong association between aberrantly expressed miRNAs and the onset and progression of CML [35,36,37]. Furthermore, miRNAs have been implicated in developing TKI resistance in CML.

Since these molecules can function as oncogenes or tumor suppressors in leukemogenesis, modulating their activity through inhibition or enhancement presents a potential therapeutic strategy, offering new avenues for leukemia treatment [35]. This study aims to investigate the altered expression patterns of miR-33a-5p and ANK3 in CML and to elucidate their potential roles in disease pathogenesis. Increasing evidence suggests that miR-33a-5p and ANK3 exhibit differential expression across various cancer types and play significant roles in cancer initiation and progression. However, the potential i*n*volvement of the miR-33a-5p/ANK3 axis in CML remains unexplored. In line with this purpose, in the present study, our data analysis showed that ANK3 mRNA expression levels were significantly decreased in CML compared to normal tissues. The exploratory pooled analysis showed a significant inverse association across K562 and HaCaT observations; however, this pattern was driven substantially by between-cell line separation and does not demonstrate co-regulation within K562 cells. These findings support further investigation of ANK3 in CML but do not establish a prognostic effect. The relationship between miR-33a-5p expression levels and CML pathogenesis was first analyzed using various bioinformatics approaches to address this gap. Subsequently, miRNA expression changes were validated through RT-qPCR. miRNet is a web-based platform designed to facilitate the exploration of miRNA functions. It enables the construction of miRNA-centered interaction networks by integrating data related to genes, diseases, small molecules, single-nucleotide polymorphisms (SNPs) that influence miRNAs or their binding sites, non-coding RNAs, epigenetic modifiers, and transcription factors [20]. This study utilized the miRNet database to identify miRNAs associated with CML. Seve*n*ty-one miRNAs were identified, and miR-33a-5p was selected for further validation analysis. The validation experiments were conducted using K562 and HaCaT cell lines, revealing that miR-33a-5p exhibited higher expression levels in K562 cells compared to HaCaT cells. Previous evidence supports a context-dependent role for miR-33a in cancer biology [14]. Sun et al., in a study on imatinib (IM)-resistant CML cell lines, demonstrated that tribbles pseudokinase 2 (TRIB2) regulates c-Fos via the ERK signaling pathway. Their findings further indicated that c-Fos suppresses transcriptional activity and miR-33a-5p expression. Additionally, TRIB2 was shown to modulate miR-33a-5p in CML to counteract IM resistance, suggesting that miR-33a-5p could serve as a novel therapeutic target for managing IM resistance in clinical treatment [38]. In another study on chronic myelomonocytic leukemia (CMML), it was found that serine/threonine kinase (PIM1) expression increased following treatment with bromodomain and extra-terminal (BET) protein inhibitors. This study further revealed that miR-33a downregulation was the underlying mechanism responsible for the upregulation of PIM1 [39].

Moreover, another study demonstrated that the overexpression of LINC01003 led to the downregulation of miR-33a-5p, thereby inhibiting multiple myeloma (MM) progression and promoting apoptosis [40]. It is well established that miRNAs regulate the expression of multiple genes involved in disease pathways and that both these molecules and their target genes hold potential as therapeutic targets [35]. miR-33a and its target genes influence various biological behaviors of tumor cells, including proliferation, metastasis, migration, invasion, cell cycle regulation, apoptosis, self-renewal, chemotherapy resistance, and radioresistance. Furthermore, they significantly impact patient outcomes, such as prognosis and overall survival [14]. This study utilized TargetScan and miRDIP databases to identify the target genes of miR-33a-5p. Among the overlapping genes identified in both databases, ANK3 was selected for further analysis. Additionally, differential gene expression data for ANK3 in CML were obtained from the GSE100026 dataset. Validation of ANK3 expression was conducted in K562 and HaCaT cells using RT-qPCR. The data extracted from the GSE100026 dataset revealed a fold-change value of −4.31 for ANK3. Furthermore, experimental findings demonstrated that ANK3 expression was lower in K562 cells compared to HaCaT cells. In the exploratory pooled analysis, miR-33a-5p and ANK3 expression were inversely associated across K562 and HaCaT observations (Spearman r_s_ = −0.836, *p* = 0.0001). Because the coefficient is strongly influenced by between-cell line separation, it should not be interpreted as evidence of co-regulation within K562 cells. The exact role of miR-33a-5p and its mechanisms in CML carcinogenesis have not been fully demonstrated. The present findings nominate a potential miR-33a-5p/ANK3 regulatory relationship in CML, but direct regulation requires functional validation. Functional enrichment analyses of miR-33a-5p-associated genes and the potential involvement of ANK3 via molecular signaling pathways in CML are not fully known.

A literature review indicates that ANK proteins are involved in various cellular processes, including intercellular connections, signal transduction, cell cycle regulation, vesicular transport, inflammatory responses, and transcriptional regulation [41]. Analysis of ANK3 expression across different tumor and normal tissues has revealed significant variations. Specifically, ANK3 was found to be significantly upregulated in chola*n*giocarcinoma and prostate adenocarcinoma. In contrast, it was markedly downregulated in tumor tissues of colon adenocarcinoma (COAD), head and neck squamous cell carcinoma, chromophobe renal cell carcinoma, clear-cell renal cell carcinoma (KIRC), and stomach adenocarcinoma. Furthermore, ANK3 expression has been linked to the immunological microenvironment, particularly in COAD, KIRC, and liver hepatocellular carcinoma [42]. Another study demonstrated that ANK3 plays a role in the PPAR signaling pathway and lipid metabolism in clear renal cell carcinoma [15].

Additionally, Kurozum et al. identified an association between ANK3, the androgen receptor (AR) signaling pathway, and breast cancer prognosis [43]. These findings suggest that ANK3 may have a potential role in cancer pathogenesis. The importance of these findings indicates that ANK3 expression affects cancer cell responses and may thus serve as a predictor for immunotherapy outcomes across various malignancies. These findings also suggest that ANK3 may be involved in cancer development. The potential functional role of ANK3 in CML was analyzed using CancerSEA. The findings indicated that ANK3 is associated with angiogenesis, apoptosis, differentiation, hypoxia, and i*n*flammation. However, further experimental research is required to fully elucidate the potential molecular mechanisms of ANK3 in CML pathogenesis and to determine whether it could serve as a therapeutic target for CML treatment. Further experimental validation should be performed to confirm the potential of ANK3 expression in relation to cancer progression, prognosis and treatment outcomes in CML, as well as immune response in the tumor microenvironment. PPI network related to ANK3 was constructed to investigate its potential molecular regulatory mechanisms. The analysis identified ANK3, CTNNB1, KRAS, CCNF, and TP53 as key genes within the PPI network. Previous studies on CML have demonstrated that CTNNB1 [44], KRAS [45,46], and TP53 [47,48] play significant roles in disease progression, aligning with the findings of this study. GO, KEGG, and Reactome pathway analyses were conducted to gain deeper insights into the pathways and biological processes influenced by these identified genes. The results revealed that these genes are significantly involved in critical pathways (*p* < 0.05), such as the Ras protein signaling pathway, regulation of the cell cycle, proteoglycans in cancer, KEGG apoptosis, and Beta-catenin independent WNT signaling, among others. Notably, previous studies have reported that Ras signaling3, cell cycle regulation, apoptotic cascades, and WNT/beta-catenin pathways play pivotal roles in CML pathogenesis. The findings align with the existing literature and support further investigation of the candidate miR-33a-5p/ANK3 relationship in CML pathophysiology. Notably, previous studies have reported that the MAPK signaling pathway [37], Ras protein signaling pathway [49], Wnt signaling pathway [50], cell cycle regulation [51], and apoptosis [52] play pivotal roles in CML pathogenesis.

The findings obtained in this study are consistent with the existing literature. Tumor cells and their surroundings generate numerous immune regulators that enhance immune suppressive factors or diminish immune activators, hence facilitating immune evasion. Previous studies demonstrated that ANK3 modulates numerous biological processes in cancer cells, encompassing cell cycle, apoptosis, and invasion [16,43]. Tan et al. discovered that ANK3 exhibits anti-angiogenic properties and is positively associated with immune agonists, which are cytokines that facilitate the differentiation and polarization of macrophages toward the M1 phenotype [42]. Bioinformatics analysis in this study also shows that it may have a beneficial effect on reconstituting the immune microenvironment for tumor suppression. Consequently, ANK3 may influence immunomodulatory gene expression or function as an immunomodulatory gene itself. The present study enhances our understanding of the potential role of the miR-33a-5p/ANK3 candidate regulatory relationship in the development and progression of CML.

However, several limitations must be acknowledged. First, most analyses conducted in this study were based on the mRNA expression levels of miR-33a-5p and ANK3. The expression relationship between miR-33a-5p and ANK3 was evaluated at the basal level. Furthermore, an additional limitation concerns the heterogeneity of the analyzed tra*n*scriptomic cohort (GSE100026), which pools samples from chronic-phase and blast-phase CML patients. While our primary in vitro cell line model, K562, is derived from CML in terminal blast crisis—making it a highly relevant and phase-appropriate model for the BP patient subset—it may not entirely capture the molecular landscape of early chro*n*ic-phase disease. Future functional and clinical studies utilizing larger, phase-stratified patient cohorts (CP vs. BP) will be crucial to clarify whether the downregulation of ANK3 and upregulation of miR-33a-5p progressively intensify during blast crisis transformation. Second, many of the conclusions drawn rely primarily on bioinformatics analyses. Vector-mediated overexpression or inhibition experiments were not conducted to observe real-time in vitro growth rates or in vivo tumor kinetics in CML models. As a result, this study lacks validation through clinical samples and biological experiments. Therefore, further fundamental and clinical research is necessary to confirm these findings and provide more comprehensive insights into the functional implications of the miR-33a-5p/ANK3 axis in CML.

## 5. Conclusions

In conclusion, miR-33a-5p expression was higher and ANK3 expression was lower in K562 cells than in HaCaT cells. Integrated target prediction, transcriptomic screening, and exploratory pooled expression analysis prioritize ANK3 as a candidate associated with miR-33a-5p. Because direct binding and functional causality were not tested, the proposed miR-33a-5p/ANK3 relationship requires validation in mechanistic and clinical studies before any therapeutic implications can be established.

## Figures and Tables

**Table 1:** GO terms enriched by ANK3, CTNNB1, KRAS, CCNF, and TP53 genes.

Category	Term	*p*-Value (Unadjusted)
BP_DIRECT	GO:0010628-positive regulation of gene expression	6.9 × 10^−5^
	GO:0060441-epithelial tube branching involved in lung morphogenesis	3.5 × 10^−3^
	GO:0014009-glial cell proliferation	3.7 × 10^−3^
	GO:0033077-T cell differentiation in thymus	8.0 × 10^−3^
	GO:0007405-neuroblast proliferation	8.6 × 10^−3^
	GO:0072089-stem cell proliferation	1.0 × 10^−2^
	GO:0043525-positive regulation of neuron apoptotic process	1.4 × 10^−2^
	GO:0007265-Ras protein signal transduction	1.7 × 10^−2^
	GO:0051402-neuron apoptotic process	1.8 × 10^−2^
	GO:0000165-MAPK cascade	2.4 × 10^−2^
	GO:0001701-in utero embryonic development	4.5 × 10^−2^
	GO:0051726-regulation of cell cycle	4.8 × 10^−2^
MF_DIRECT	GO:0044325-transmembrane transporter binding	3.0 × 10^−2^
	GO:0061629-RNA polymerase II-specific DNA-binding transcription factor binding	3.8 × 10^−2^

Gene Ontology (GO), Molecular Function (MF), Biological Process (BP).

**Table 2:** KEGG and Reactome pathway enrichment analyses of ANK3, TP53, CCNF, KRAS, and CTNNB1.

Category	Term	Count	%	*p*-Value (Unadjusted)
KEGG PATHWAY	Proteoglycans in cancer	4	80	1.2 × 10^−5^
	Thyroid cancer	3	60	5.1 × 10^−5^
	Endometrial cancer	3	60	1.3 × 10^−4^
	Colorectal cancer	3	60	2.8 × 10^−4^
	Prostate cancer	3	60	3.6 × 10^−4^
	Thyroid hormone signaling pathway	3	60	5.6 × 10^−4^
	Breast cancer	3	60	8.2 × 10^−4^
	Gastric cancer	3	60	8.4 × 10^−4^
	Hepatitis C	3	60	9.5 × 10^−4^
	Hepatocellular carcinoma	3	60	1.1 × 10^−3^
	Kaposi sarcoma-associated herpesvirus infection	3	60	1.4 × 10^−3^
	Human cytomegalovirus infection	3	60	1.9 × 10^−3^
	Human papillomavirus infection	3	60	4.1 × 10^−3^
	Pathways in cancer	3	60	1.0 × 10^−2^
	Bladder cancer	2	40	1.4 × 10^−2^
	Basal cell carcinoma	2	40	2.1 × 10^−2^
	Central carbon metabolism in cancer	2	40	2.4 × 10^−2^
	Melanoma	2	40	2.5 × 10^−2^
	Non-small-cell lung cancer	2	40	2.5 × 10^−2^
	Glioma	2	40	2.6 × 10^−2^
	Chronic myeloid leukemia	2	40	2.6 × 10^−2^
	Pancreatic cancer	2	40	2.6 × 10^−2^
	Longevity regulating pathway	2	40	3.0 × 10^−2^
	Endocrine resistance	2	40	3.3 × 10^−2^
	Melanogenesis	2	40	3.4 × 10^−2^
	Mitophagy—animal	2	40	3.5 × 10^−2^
	Neurotrophin signaling pathway	2	40	4.0 × 10^−2^
	Sphingolipid signaling pathway	2	40	4.1 × 10^−2^
	Apoptosis	2	40	4.5 × 10^−2^
	Fluid shear stress and atherosclerosis	2	40	4.7 × 10^−2^
	Signaling pathways regulating pluripotency of stem cells	2	40	4.8 × 10^−2^
REACTOMEPATHWAY	Transcriptional regulation by RUNX3	3	60	3.5 × 10^−4^
	Developmental Biology	4	80	7.1 × 10^−3^
	Diseases of signal transduction by growth factor receptors and second messengers	3	60	9.3 × 10^−3^
	Transcriptional Regulation by VENTX	2	40	1.4 × 10^−2^
	Immune System	4	80	2.1 × 10^−2^
	Ca^2+^ pathway	2	40	2.2 × 10^−2^
	Metabolism of proteins	4	80	2.3 × 10^−2^
	VEGFA-VEGFR2 Pathway	2	40	3.5 × 10^−2^
	Signaling by VEGF	2	40	3.8 × 10^−2^
	Beta-catenin independent WNT signaling	2	40	4.7 × 10^−2^
	Innate Immune System	3	60	4.7 × 10^−2^

Count: enriched gene number in the KEGG and Reactome analyses.

## Data Availability

The original data presented in the study are openly available in the NCBI Gene Expression Omnibus (GEO) at https://www.ncbi.nlm.nih.gov/geo/query/acc.cgi?acc=GSE100026, reference number GSE100026.

## Supplementary Materials

The following supporting information can be downloaded at: https://www.mdpi.com/article/10.3390/cimb48090949/s1. Table S1: miR-33a-5p targets obtained from TargetScan; Table S2: miR-33a-5p targets obtained from mirDIP.

## Abbreviations

CML	Chronic myeloid leukemia
ANK3	Ankyrin 3
GAPDH	Glyceraldehyde 3-phosphate dehydrogenase
TKIs	Tyrosine kinase inhibitors
AR	Androgen receptor
