# Supplementary material for: Candidate Regulatory Relationship and Expression Correlation Between miR-33a-5p and ANK3 in Chronic Myeloid Leukemia
Source: Curr Issues Mol Biol. 2026 Sep 17;48(9):949. doi: 10.3390/cimb48090949 (PMC13605770; doi:10.3390/cimb48090949)
Supplement: Supplementary file 1 [file cimb-48-00949-s001.zip › cimb-4533517-supplementary.pdf]

Table S1: miR-33a-5p targets obtained from TargetScan.

Target gene	Representative miRNA
ANO4	hsa-miR-33a-5p
UBE2V2	hsa-miR-33a-5p
CROT	hsa-miR-33a-5p
CCNYL1	hsa-miR-33a-5p
EN2	hsa-miR-33a-5p
NPC1	hsa-miR-33a-5p
RCAN1	hsa-miR-33a-5p
SIRT6	hsa-miR-33a-5p
YWHAH	hsa-miR-33a-5p
CSMD1	hsa-miR-33a-5p
SLC25A25	hsa-miR-33a-5p
GLCCI1	hsa-miR-33a-5p
AL354993.1	hsa-miR-33a-5p
ANKRD44	hsa-miR-33a-5p
PIM1	hsa-miR-33a-5p
NRXN1	hsa-miR-33a-5p
SNAI2	hsa-miR-33a-5p
RORA	hsa-miR-33a-5p
RP11-113D6.10	hsa-miR-33a-5p
BEND6	hsa-miR-33a-5p
ZNF281	hsa-miR-33a-5p
LDHA	hsa-miR-33a-5p
ZNF804B	hsa-miR-33a-5p
HADHB	hsa-miR-33a-5p
KIAA0087	hsa-miR-33a-5p
DEPTOR	hsa-miR-33a-5p
SLC17A6	hsa-miR-33a-5p
ABCA1	hsa-miR-33a-5p
SREK1	hsa-miR-33a-5p
GAS1	hsa-miR-33a-5p
SPAST	hsa-miR-33a-5p
TM9SF3	hsa-miR-33a-5p
EIF5A2	hsa-miR-33a-5p
FAM222B	hsa-miR-33a-5p
CDK6	hsa-miR-33a-5p
GRIK2	hsa-miR-33a-5p
CPT1A	hsa-miR-33a-5p
NSF	hsa-miR-33a-5p
RGMA	hsa-miR-33a-5p
ITGBL1	hsa-miR-33a-5p
APPBP2	hsa-miR-33a-5p
BACH1	hsa-miR-33a-5p
TMEM59	hsa-miR-33a-5p

KIAA2026	hsa-miR-33a-5p
CPSF2	hsa-miR-33a-5p
ZC3H12C	hsa-miR-33a-5p
MAPK8	hsa-miR-33a-5p
ABHD2	hsa-miR-33a-5p
TMEM86A	hsa-miR-33a-5p
PIM3	hsa-miR-33a-5p
RNF128	hsa-miR-33a-5p
ANO8	hsa-miR-33a-5p
MAP3K7	hsa-miR-33a-5p
RAP2A	hsa-miR-33a-5p
ESCO1	hsa-miR-33a-5p
EEF1A1	hsa-miR-33a-5p
EBAG9	hsa-miR-33a-5p
DCUN1D5	hsa-miR-33a-5p
UNC79	hsa-miR-33a-5p
SELT	hsa-miR-33a-5p
GPR88	hsa-miR-33a-5p
SALL1	hsa-miR-33a-5p
CRTAM	hsa-miR-33a-5p
MCFD2	hsa-miR-33a-5p
TTC28	hsa-miR-33a-5p
DDX5	hsa-miR-33a-5p
EGFLAM	hsa-miR-33a-5p
SMARCA5	hsa-miR-33a-5p
NAA15	hsa-miR-33a-5p
RNF115	hsa-miR-33a-5p
NIPAL4	hsa-miR-33a-5p
SGIP1	hsa-miR-33a-5p
TMED5	hsa-miR-33a-5p
AC007405.2	hsa-miR-33a-5p
THAP1	hsa-miR-33a-5p
SATB2	hsa-miR-33a-5p
RASSF8	hsa-miR-33a-5p
UNCX	hsa-miR-33a-5p
SNX9	hsa-miR-33a-5p
SETD9	hsa-miR-33a-5p
CACNA1C	hsa-miR-33a-5p
METTL9	hsa-miR-33a-5p
CAMK2N1	hsa-miR-33a-5p
ISOC2	hsa-miR-33a-5p
ST3GAL2	hsa-miR-33a-5p
CAMK4	hsa-miR-33a-5p
NHLH2	hsa-miR-33a-5p
ATP8B1	hsa-miR-33a-5p
SEC24C	hsa-miR-33a-5p
CELF2	hsa-miR-33a-5p
NUTF2	hsa-miR-33a-5p

STT3B	hsa-miR-33a-5p
ABCB5	hsa-miR-33a-5p
HMGN1	hsa-miR-33a-5p
GRM8	hsa-miR-33a-5p
SHISA5	hsa-miR-33a-5p
CCNY	hsa-miR-33a-5p
EPHA8	hsa-miR-33a-5p
C8orf86	hsa-miR-33a-5p
RASEF	hsa-miR-33a-5p
HAS3	hsa-miR-33a-5p
LUC7L3	hsa-miR-33a-5p
RBM33	hsa-miR-33a-5p
CEND1	hsa-miR-33a-5p
RMND5A	hsa-miR-33a-5p
DNAJB5	hsa-miR-33a-5p
HOXD10	hsa-miR-33a-5p
BMP3	hsa-miR-33a-5p
GPR37	hsa-miR-33a-5p
PFN2	hsa-miR-33a-5p
EIF2S2	hsa-miR-33a-5p
DKK1	hsa-miR-33a-5p
RNF180	hsa-miR-33a-5p
BLOC1S5	hsa-miR-33a-5p
HMGCR	hsa-miR-33a-5p
HOXC13	hsa-miR-33a-5p
LRRC4	hsa-miR-33a-5p
MRPL49	hsa-miR-33a-5p
MTHFD2	hsa-miR-33a-5p
PAPOLG	hsa-miR-33a-5p
CDC42BPA	hsa-miR-33a-5p
DSC3	hsa-miR-33a-5p
AKAP2	hsa-miR-33a-5p
FST	hsa-miR-33a-5p
CS	hsa-miR-33a-5p
IMPA2	hsa-miR-33a-5p
CRK	hsa-miR-33a-5p
CSNK1D	hsa-miR-33a-5p
RECK	hsa-miR-33a-5p
KIAA1211L	hsa-miR-33a-5p
USP46	hsa-miR-33a-5p
UNK	hsa-miR-33a-5p
DUSP6	hsa-miR-33a-5p
SMAD7	hsa-miR-33a-5p
CALN1	hsa-miR-33a-5p
STS	hsa-miR-33a-5p
SKP1	hsa-miR-33a-5p
RERG	hsa-miR-33a-5p
MBOAT2	hsa-miR-33a-5p

SPIN1	hsa-miR-33a-5p
FYN	hsa-miR-33a-5p
RASA1	hsa-miR-33a-5p
ARID5B	hsa-miR-33a-5p
KCNJ1	hsa-miR-33a-5p
PALM2-AKAP2	hsa-miR-33a-5p
CCDC85A	hsa-miR-33a-5p
TMEM200B	hsa-miR-33a-5p
PPTC7	hsa-miR-33a-5p
TMED7	hsa-miR-33a-5p
ALKBH1	hsa-miR-33a-5p
TRIM6	hsa-miR-33a-5p
EBF1	hsa-miR-33a-5p
TSPAN9	hsa-miR-33a-5p
SLC12A5	hsa-miR-33a-5p
WDR47	hsa-miR-33a-5p
ZNF496	hsa-miR-33a-5p
PCDH10	hsa-miR-33a-5p
NTN1	hsa-miR-33a-5p
ZFPM2	hsa-miR-33a-5p
FAM84A	hsa-miR-33a-5p
HS3ST4	hsa-miR-33a-5p
ZFP36L1	hsa-miR-33a-5p
ADO	hsa-miR-33a-5p
BTBD2	hsa-miR-33a-5p
BAI3	hsa-miR-33a-5p
COLCA1	hsa-miR-33a-5p
NCOA7	hsa-miR-33a-5p
TPM3	hsa-miR-33a-5p
TES	hsa-miR-33a-5p
KPNA4	hsa-miR-33a-5p
HNRNPA3	hsa-miR-33a-5p
SKI	hsa-miR-33a-5p
FAM160A1	hsa-miR-33a-5p
NCOA3	hsa-miR-33a-5p
PANK3	hsa-miR-33a-5p
NCOA2	hsa-miR-33a-5p
REEP1	hsa-miR-33a-5p
KCND3	hsa-miR-33a-5p
AHCYL1	hsa-miR-33a-5p
ADGB	hsa-miR-33a-5p
PCDH18	hsa-miR-33a-5p
NAA30	hsa-miR-33a-5p
BTBD3	hsa-miR-33a-5p
KCNK1	hsa-miR-33a-5p
CTTNBP2	hsa-miR-33a-5p
TBX15	hsa-miR-33a-5p
MAPK6	hsa-miR-33a-5p

NAALADL2	hsa-miR-33a-5p
KCTD1	hsa-miR-33a-5p
SEPT7	hsa-miR-33a-5p
NEUROG2	hsa-miR-33a-5p
ROCK1	hsa-miR-33a-5p
DACH1	hsa-miR-33a-5p
AC012215.1	hsa-miR-33a-5p
TSHZ1	hsa-miR-33a-5p
SYT11	hsa-miR-33a-5p
OXSR1	hsa-miR-33a-5p
SLC6A5	hsa-miR-33a-5p
ALX4	hsa-miR-33a-5p
NDNF	hsa-miR-33a-5p
FBXO34	hsa-miR-33a-5p
SLC30A4	hsa-miR-33a-5p
TSHZ2	hsa-miR-33a-5p
TSC22D2	hsa-miR-33a-5p
PIAS4	hsa-miR-33a-5p
MDGA2	hsa-miR-33a-5p
CLGN	hsa-miR-33a-5p
HIPK1	hsa-miR-33a-5p
ABR	hsa-miR-33a-5p
KLHL15	hsa-miR-33a-5p
SHISA7	hsa-miR-33a-5p
TRAM2	hsa-miR-33a-5p
ROBO2	hsa-miR-33a-5p
MACROD2	hsa-miR-33a-5p
TRAF3	hsa-miR-33a-5p
FAM81A	hsa-miR-33a-5p
NRIP1	hsa-miR-33a-5p
GALNT10	hsa-miR-33a-5p
PRICKLE2	hsa-miR-33a-5p
ZNF385B	hsa-miR-33a-5p
TMEM260	hsa-miR-33a-5p
RAB30	hsa-miR-33a-5p
CDK8	hsa-miR-33a-5p
MTF1	hsa-miR-33a-5p
ZMIZ1	hsa-miR-33a-5p
LHX6	hsa-miR-33a-5p
DPP10	hsa-miR-33a-5p
PAPOLA	hsa-miR-33a-5p
HSPA8	hsa-miR-33a-5p
CADM2	hsa-miR-33a-5p
DCUN1D1	hsa-miR-33a-5p
PCNX	hsa-miR-33a-5p
KIAA1549L	hsa-miR-33a-5p
BACH2	hsa-miR-33a-5p
ADAMTS3	hsa-miR-33a-5p

PCDHA7	hsa-miR-33a-5p
PCDHA1	hsa-miR-33a-5p
PCDHA9	hsa-miR-33a-5p
PCDHA10	hsa-miR-33a-5p
PCDHA6	hsa-miR-33a-5p
PCDHA12	hsa-miR-33a-5p
PCDHA5	hsa-miR-33a-5p
PCDHAC1	hsa-miR-33a-5p
PCDHA3	hsa-miR-33a-5p
PCDHA8	hsa-miR-33a-5p
PCDHA13	hsa-miR-33a-5p
PCDHA2	hsa-miR-33a-5p
PCDHA11	hsa-miR-33a-5p
PCDHA4	hsa-miR-33a-5p
CPEB2	hsa-miR-33a-5p
TARDBP	hsa-miR-33a-5p
CHN2	hsa-miR-33a-5p
RGS17	hsa-miR-33a-5p
GABRB2	hsa-miR-33a-5p
PCDHAC2	hsa-miR-33a-5p
GDNF	hsa-miR-33a-5p
SLITRK3	hsa-miR-33a-5p
PSME3	hsa-miR-33a-5p
WNT10B	hsa-miR-33a-5p
PDPK1	hsa-miR-33a-5p
PIK3R3	hsa-miR-33a-5p
RORB	hsa-miR-33a-5p
TERF2	hsa-miR-33a-5p
UBR5	hsa-miR-33a-5p
TBC1D15	hsa-miR-33a-5p
KCNJ13	hsa-miR-33a-5p
ONECUT2	hsa-miR-33a-5p
ZSWIM6	hsa-miR-33a-5p
KMT2E	hsa-miR-33a-5p
NLGN1	hsa-miR-33a-5p
FGF14	hsa-miR-33a-5p
DST	hsa-miR-33a-5p
PDE8A	hsa-miR-33a-5p
TRPC4	hsa-miR-33a-5p
SAMD8	hsa-miR-33a-5p
TET2	hsa-miR-33a-5p
VCL	hsa-miR-33a-5p
ADCYAP1	hsa-miR-33a-5p
ABHD13	hsa-miR-33a-5p
EDNRB	hsa-miR-33a-5p
GDI2	hsa-miR-33a-5p
ERBB2IP	hsa-miR-33a-5p
RPGRIP1L	hsa-miR-33a-5p

EVI5	hsa-miR-33a-5p
ADD3	hsa-miR-33a-5p
ANK3	hsa-miR-33a-5p
HAS2	hsa-miR-33a-5p
ESRRG	hsa-miR-33a-5p
PDIK1L	hsa-miR-33a-5p
SAMD4A	hsa-miR-33a-5p
S1PR1	hsa-miR-33a-5p
MAP3K1	hsa-miR-33a-5p
SH3KBP1	hsa-miR-33a-5p
LDLRAD4	hsa-miR-33a-5p
SETD7	hsa-miR-33a-5p
DENND5B	hsa-miR-33a-5p
KLF7	hsa-miR-33a-5p
NFIX	hsa-miR-33a-5p
HIPK2	hsa-miR-33a-5p
KIAA1024	hsa-miR-33a-5p
CPEB3	hsa-miR-33a-5p
LARP4B	hsa-miR-33a-5p
NCOA1	hsa-miR-33a-5p
ADRA2A	hsa-miR-33a-5p
LIN28A	hsa-miR-33a-5p
USP6	hsa-miR-33a-5p
RNF19B	hsa-miR-33a-5p
MRS2	hsa-miR-33a-5p
DGKH	hsa-miR-33a-5p
AGMAT	hsa-miR-33a-5p
MEIS1	hsa-miR-33a-5p
DCAF8	hsa-miR-33a-5p
CDR2L	hsa-miR-33a-5p
SCN8A	hsa-miR-33a-5p
RBPMS2	hsa-miR-33a-5p
ZDHHC3	hsa-miR-33a-5p
SEL1L3	hsa-miR-33a-5p
PHF12	hsa-miR-33a-5p
EPC2	hsa-miR-33a-5p
PLD5	hsa-miR-33a-5p
NOMO2	hsa-miR-33a-5p
PDE4D	hsa-miR-33a-5p
ENC1	hsa-miR-33a-5p
PURB	hsa-miR-33a-5p
TFDP2	hsa-miR-33a-5p
NOMO1	hsa-miR-33a-5p
PPP6C	hsa-miR-33a-5p
SH2B3	hsa-miR-33a-5p
HECW1	hsa-miR-33a-5p
TRPM3	hsa-miR-33a-5p
NR4A2	hsa-miR-33a-5p

FRS2	hsa-miR-33a-5p
MAP4K4	hsa-miR-33a-5p
C2orf69	hsa-miR-33a-5p
PDGFRA	hsa-miR-33a-5p
CSRNP3	hsa-miR-33a-5p
MSRB3	hsa-miR-33a-5p
NFYB	hsa-miR-33a-5p
RFX3	hsa-miR-33a-5p
AFF3	hsa-miR-33a-5p
SYDE2	hsa-miR-33a-5p
ERC1	hsa-miR-33a-5p
ZNF24	hsa-miR-33a-5p
CREBBP	hsa-miR-33a-5p
SOCS5	hsa-miR-33a-5p
LPPR4	hsa-miR-33a-5p
PRKAA1	hsa-miR-33a-5p
FAM46C	hsa-miR-33a-5p
EPHA4	hsa-miR-33a-5p
GRID2	hsa-miR-33a-5p
SMG1	hsa-miR-33a-5p
CNOT2	hsa-miR-33a-5p
BAG2	hsa-miR-33a-5p
CREBRF	hsa-miR-33a-5p
ZNF207	hsa-miR-33a-5p
CACNA1D	hsa-miR-33a-5p
SNRK	hsa-miR-33a-5p
KCNS2	hsa-miR-33a-5p
QKI	hsa-miR-33a-5p
SNTB2	hsa-miR-33a-5p
ST18	hsa-miR-33a-5p
FAM179B	hsa-miR-33a-5p
BCL11B	hsa-miR-33a-5p
NUFIP2	hsa-miR-33a-5p
HMX3	hsa-miR-33a-5p
RIMBP2	hsa-miR-33a-5p
CAMK2G	hsa-miR-33a-5p
PIP4K2C	hsa-miR-33a-5p
CERK	hsa-miR-33a-5p
GRIN3A	hsa-miR-33a-5p
NFIA	hsa-miR-33a-5p
FAM126B	hsa-miR-33a-5p
MAP3K3	hsa-miR-33a-5p
TTBK2	hsa-miR-33a-5p
SCN10A	hsa-miR-33a-5p
REEP3	hsa-miR-33a-5p
HCN1	hsa-miR-33a-5p
ATXN7L1	hsa-miR-33a-5p
CDK16	hsa-miR-33a-5p

ST8SIA3	hsa-miR-33a-5p
TCF4	hsa-miR-33a-5p
NAP1L4	hsa-miR-33a-5p
CTBP2	hsa-miR-33a-5p
PLEKHA8	hsa-miR-33a-5p
SIM2	hsa-miR-33a-5p
VCAN	hsa-miR-33a-5p
DDX46	hsa-miR-33a-5p
DESI1	hsa-miR-33a-5p
CLPX	hsa-miR-33a-5p
DAB2IP	hsa-miR-33a-5p
TNRC6C	hsa-miR-33a-5p
PDE3B	hsa-miR-33a-5p
GHR	hsa-miR-33a-5p
DLGAP2	hsa-miR-33a-5p
KAT6B	hsa-miR-33a-5p
NAV3	hsa-miR-33a-5p
GSE1	hsa-miR-33a-5p
IRS2	hsa-miR-33a-5p
SLC35E1	hsa-miR-33a-5p
SREBF1	hsa-miR-33a-5p
SIK1	hsa-miR-33a-5p
KIAA1432	hsa-miR-33a-5p
ASXL2	hsa-miR-33a-5p
SCN1A	hsa-miR-33a-5p
ONECUT3	hsa-miR-33a-5p
LINGO1	hsa-miR-33a-5p
PTBP3	hsa-miR-33a-5p
SGCD	hsa-miR-33a-5p
MT1A	hsa-miR-33a-5p
SYT14	hsa-miR-33a-5p
RLIM	hsa-miR-33a-5p
DCP1A	hsa-miR-33a-5p
CAPZB	hsa-miR-33a-5p
PCDH7	hsa-miR-33a-5p
USP32	hsa-miR-33a-5p
TRPS1	hsa-miR-33a-5p
RNF165	hsa-miR-33a-5p
CREBZF	hsa-miR-33a-5p
CDYL2	hsa-miR-33a-5p
ATP1B1	hsa-miR-33a-5p
MYPN	hsa-miR-33a-5p
LAMC1	hsa-miR-33a-5p
SLC16A7	hsa-miR-33a-5p
HNRNPA2B1	hsa-miR-33a-5p
PTGFRN	hsa-miR-33a-5p
SLC24A4	hsa-miR-33a-5p
CACNA1E	hsa-miR-33a-5p

GIGYF1	hsa-miR-33a-5p
NOVA1	hsa-miR-33a-5p
CNNM1	hsa-miR-33a-5p
RUNX1T1	hsa-miR-33a-5p
CCDC144A	hsa-miR-33a-5p
PACSIN1	hsa-miR-33a-5p
KCNC3	hsa-miR-33a-5p
CASC3	hsa-miR-33a-5p
MTDH	hsa-miR-33a-5p
CPEB4	hsa-miR-33a-5p
CTDSPL2	hsa-miR-33a-5p
PNMA2	hsa-miR-33a-5p
KIF1B	hsa-miR-33a-5p
CNTN4	hsa-miR-33a-5p
AP5M1	hsa-miR-33a-5p
TMEM65	hsa-miR-33a-5p
ZFHX4	hsa-miR-33a-5p
NPAT	hsa-miR-33a-5p
ATP8A1	hsa-miR-33a-5p
ZCCHC14	hsa-miR-33a-5p
DDX3X	hsa-miR-33a-5p
PJA2	hsa-miR-33a-5p
KIAA0907	hsa-miR-33a-5p
AKAP12	hsa-miR-33a-5p
FOXP1	hsa-miR-33a-5p
XBP1	hsa-miR-33a-5p
C18orf25	hsa-miR-33a-5p
TNS4	hsa-miR-33a-5p
MLLT3	hsa-miR-33a-5p
TANC2	hsa-miR-33a-5p
PDPN	hsa-miR-33a-5p
SEMA7A	hsa-miR-33a-5p
STRN	hsa-miR-33a-5p
CHD1	hsa-miR-33a-5p
SRGAP1	hsa-miR-33a-5p
KPNA3	hsa-miR-33a-5p
ADD1	hsa-miR-33a-5p
BRWD3	hsa-miR-33a-5p
ZBTB46	hsa-miR-33a-5p
SUV39H1	hsa-miR-33a-5p
FAM120A	hsa-miR-33a-5p
PAQR3	hsa-miR-33a-5p
GPCPD1	hsa-miR-33a-5p
RFX7	hsa-miR-33a-5p
SCIN	hsa-miR-33a-5p
RIMS2	hsa-miR-33a-5p
ALPK3	hsa-miR-33a-5p
YTHDF3	hsa-miR-33a-5p

KMT2D	hsa-miR-33a-5p
MYT1L	hsa-miR-33a-5p
AXL	hsa-miR-33a-5p
TTLL7	hsa-miR-33a-5p
SPPL3	hsa-miR-33a-5p
CD302	hsa-miR-33a-5p
CDH6	hsa-miR-33a-5p
PLEKHA3	hsa-miR-33a-5p
ST3GAL6	hsa-miR-33a-5p
PPP1R9A	hsa-miR-33a-5p
KCTD16	hsa-miR-33a-5p
TULP4	hsa-miR-33a-5p
PDE3A	hsa-miR-33a-5p
UNC5D	hsa-miR-33a-5p
PSMG1	hsa-miR-33a-5p
FGF12	hsa-miR-33a-5p
WDFY3	hsa-miR-33a-5p
PDZD8	hsa-miR-33a-5p
PLEKHM3	hsa-miR-33a-5p
TIMM9	hsa-miR-33a-5p
SDC3	hsa-miR-33a-5p
TBCEL	hsa-miR-33a-5p
SESTD1	hsa-miR-33a-5p
ZNF324	hsa-miR-33a-5p
SHANK2	hsa-miR-33a-5p
SBNO1	hsa-miR-33a-5p
STXBP5	hsa-miR-33a-5p
TFCP2L1	hsa-miR-33a-5p
NDUFB9	hsa-miR-33a-5p
EHD4	hsa-miR-33a-5p
RND2	hsa-miR-33a-5p
DNAJC15	hsa-miR-33a-5p
STAU2	hsa-miR-33a-5p
LY75-CD302	hsa-miR-33a-5p
GBX2	hsa-miR-33a-5p
CYB5R4	hsa-miR-33a-5p
ABI2	hsa-miR-33a-5p
HUNK	hsa-miR-33a-5p
RAB41	hsa-miR-33a-5p
LRRC4C	hsa-miR-33a-5p
ABCB11	hsa-miR-33a-5p
NCKAP1	hsa-miR-33a-5p
FTO	hsa-miR-33a-5p
POFUT2	hsa-miR-33a-5p
PPL	hsa-miR-33a-5p
MORF4L1	hsa-miR-33a-5p
IQSEC2	hsa-miR-33a-5p
KCNK15	hsa-miR-33a-5p

Table S2: miR-33a-5p targets obtained from mirDIP

inks	Gene Symbol	Uniprot	Pseudogene	microRNA	Integrated Score	Number of Sources	Score Class
GC UP	**HADHB**	P55084		hsa-miR-33a-5p	0.864507754097481	18	Very High
GC UP	**ABHD2**	P08910		hsa-miR-33a-5p	0.851397751149634	18	Very High
GC UP	**ATP1B1**	P05026		hsa-miR-33a-5p	0.806982471732787	18	Very High
GC UP	**CROT**	Q9UKG9		hsa-miR-33a-5p	0.929778645398148	17	Very High
GC UP	**ABCA1**	O95477		hsa-miR-33a-5p	0.891221858171468	17	Very High
GC UP	**CDK6**	Q00534		hsa-miR-33a-5p	0.870460059901457	17	Very High
GC UP	**SATB2**	Q9UPW6		hsa-miR-33a-5p	0.862154583412539	17	Very High
GC UP	**NAA15**	Q9BXJ9		hsa-miR-33a-5p	0.83952883263945	17	Very High
GC UP	**KPNA4**	O00629		hsa-miR-33a-5p	0.798359612120297	17	Very High
GC UP	**MLLT3**	P42568		hsa-miR-33a-5p	0.786738573423489	17	Very High
GC UP	**ROBO2**	Q9HCK4		hsa-miR-33a-5p	0.776836719538308	17	Very High
GC UP	**MAP3K3**	Q99759		hsa-miR-33a-5p	0.733691997921068	17	Very High
GC UP	**ATP8B1**	O43520		hsa-miR-33a-5p	0.829865341921343	16	Very High
GC UP	**RAP2A**	P10114		hsa-miR-33a-5p	0.819148468755122	16	Very High
GC UP	**APPBP2**	Q92624		hsa-miR-33a-5p	0.792564907278871	16	Very High
GC UP	**PDGFRA**	P16234		hsa-miR-33a-5p	0.792305506885208	16	Very High
GC UP	**RNF128**	Q8TEB7		hsa-miR-33a-5p	0.789026394332008	16	Very High
GC UP	**PRICKLE2**	Q7Z3G6		hsa-miR-33a-5p	0.773376152902199	16	Very High
GC UP	**GDI2**	P50395		hsa-miR-33a-5p	0.773203687568636	16	Very High
GC UP	**ZEB2**	O60315		hsa-miR-33a-5p	0.717050382043421	16	Very High
GC UP	**RASSF8**	Q8NHQ8		hsa-miR-33a-5p	0.709048238679114	16	Very High
GC UP	**HMGA2**	P52926		hsa-miR-33a-5p	0.899390315496692	15	Very High
GC UP	**GLCCI1**	Q86VQ1		hsa-miR-33a-5p	0.865595245242669	15	Very High
GC UP	**EN2**	P19622		hsa-miR-33a-5p	0.860155976997362	15	Very High
GC UP	**SPAST**	Q9UBP0		hsa-miR-33a-5p	0.818815065616437	15	Very High
GC UP	**CACNA1C**	Q13936		hsa-miR-33a-5p	0.81179290842532	15	Very High
GC UP	**BACH1**	O14867		hsa-miR-33a-5p	0.793534978688673	15	Very High
GC UP	**DDX5**	P17844		hsa-miR-33a-5p	0.791582386998065	15	Very High
GC UP	**CDC42BPA**	Q5VT25		hsa-miR-33a-5p	0.790168915078018	15	Very High
GC UP	**TTC28**	Q96AY4		hsa-miR-33a-5p	0.787855199748332	15	Very High
GC UP	**B3GALT2**	O43825		hsa-miR-33a-5p	0.785264616211337	15	Very High
GC UP	**MORF4L1**	Q9UBU8		hsa-miR-33a-5p	0.777525558259688	15	Very High

GC	UP	**HNRNPA2B1**	P22626	hsa-miR-33a-5p	0.774486911741899	15	Very High
GC	UP	**HIPK1**	Q86Z02	hsa-miR-33a-5p	0.773882284622674	15	Very High
GC	UP	**EBF1**	Q9UH73	hsa-miR-33a-5p	0.770459151948467	15	Very High
GC	UP	**SNRK**	Q9NRH2	hsa-miR-33a-5p	0.767914424120509	15	Very High
GC	UP	**SETD7**	Q8WTS6	hsa-miR-33a-5p	0.763521719068986	15	Very High
GC	UP	**LUC7L3**	O95232	hsa-miR-33a-5p	0.761440953228806	15	Very High
GC	UP	**NAA30**	Q147X3	hsa-miR-33a-5p	0.754626314328193	15	Very High
GC	UP	**CAND1**	Q86VP6	hsa-miR-33a-5p	0.743701851305032	15	Very High
GC	UP	**ASAP1**	Q9ULH1	hsa-miR-33a-5p	0.743680097998649	15	Very High
GC	UP	**KIF1B**	O60333	hsa-miR-33a-5p	0.731979847116359	15	Very High
GC	UP	**PCDH7**	O60245	hsa-miR-33a-5p	0.730622115534929	15	Very High
GC	UP	**SMG1**	Q96Q15	hsa-miR-33a-5p	0.727767667109454	15	Very High
GC	UP	**CSNK1D**	P48730	hsa-miR-33a-5p	0.708953191500908	15	Very High
GC	UP	**CAMK2G**	Q13555	hsa-miR-33a-5p	0.699054485659483	15	Very High
GC	UP	**VCPIP1**	Q96JH7	hsa-miR-33a-5p	0.693632599414518	15	Very High
GC	UP	**YWHAH**	Q04917	hsa-miR-33a-5p	0.890841486807731	14	Very High
GC	UP	**UBE2V2**	Q15819	hsa-miR-33a-5p	0.860537563251534	14	Very High
GC	UP	**ZNF281**	Q9Y2X9	hsa-miR-33a-5p	0.842039455712036	14	Very High
GC	UP	**SLC25A25**	Q6KCM7	hsa-miR-33a-5p	0.837445583609236	14	Very High
GC	UP	**SREK1**	Q8WXA9	hsa-miR-33a-5p	0.809981065469173	14	Very High
GC	UP	**ARID5B**	Q14865	hsa-miR-33a-5p	0.805151150887081	14	Very High
GC	UP	**MAP4K4**	O95819	hsa-miR-33a-5p	0.798756023035201	14	Very High
GC	UP	**TM9SF3**	Q9HD45	hsa-miR-33a-5p	0.7873444831475	14	Very High
GC	UP	**SMARCA5**	O60264	hsa-miR-33a-5p	0.782829644338753	14	Very High
GC	UP	**BACH2**	Q9BYV9	hsa-miR-33a-5p	0.777555186975869	14	Very High
GC	UP	**RCAN1**	P53805	hsa-miR-33a-5p	0.768914575879005	14	Very High
GC	UP	**CPT1A**	P50416	hsa-miR-33a-5p	0.768357035220475	14	Very High
GC	UP	**SLC12A5**	Q9H2X9	hsa-miR-33a-5p	0.766649802010155	14	Very High
GC	UP	**HIPK2**	Q9H2X6	hsa-miR-33a-5p	0.762404358971847	14	Very High
GC	UP	**EIF5A2**	Q9GZV4	hsa-miR-33a-5p	0.761956650068967	14	Very High
GC	UP	**PAPOLG**	Q9BWT3	hsa-miR-33a-5p	0.755843444925376	14	Very High
GC	UP	**LDLRAD4**	O15165	hsa-miR-33a-5p	0.753827092319159	14	Very High
GC	UP	**CPEB3**	Q8NE35	hsa-miR-33a-5p	0.753089382231232	14	Very High
GC	UP	**PRKAA1**	Q13131	hsa-miR-33a-5p	0.752737512589812	14	Very High
GC	UP	**MTF1**	Q14872	hsa-miR-33a-5p	0.752249447711299	14	Very High
GC	UP	**RMND5A**	Q9H871	hsa-miR-33a-5p	0.751306623507608	14	Very High
GC	UP	**EEF1A1**	P68104	hsa-miR-33a-5p	0.748303038414375	14	Very High
GC	UP	**SHANK2**	Q9UPX8	hsa-miR-33a-5p	0.74738541923276	14	Very High

GC	UP	**MMP16**	P51512	hsa-miR-33a-5p	0.743189125253932	14	Very High
GC	UP	**SLC17A6**	Q9P2U8	hsa-miR-33a-5p	0.743167327080996	14	Very High
GC	UP	**GRIA3**	P42263	hsa-miR-33a-5p	0.74215881168965	14	Very High
GC	UP	**CLPX**	O76031	hsa-miR-33a-5p	0.740164029851784	14	Very High
GC	UP	**SPIN1**	Q9Y657	hsa-miR-33a-5p	0.739355427938901	14	Very High
GC	UP	**SIX4**	Q9UIU6	hsa-miR-33a-5p	0.737557946577087	14	Very High
GC	UP	**ANK3**	Q12955	hsa-miR-33a-5p	0.736647994747758	14	Very High
GC	UP	**WDFY3**	Q8IZQ1	hsa-miR-33a-5p	0.73498284210287	14	Very High
GC	UP	**MORF4L2**	Q15014	hsa-miR-33a-5p	0.731282609921114	14	Very High
GC	UP	**UBR5**	O95071	hsa-miR-33a-5p	0.730227170888267	14	Very High
GC	UP	**CAMK4**	Q16566	hsa-miR-33a-5p	0.729741168499872	14	Very High
GC	UP	**USP32**	Q8NFA0	hsa-miR-33a-5p	0.729578096676975	14	Very High
GC	UP	**TANC2**	Q9HCD6	hsa-miR-33a-5p	0.723065001141256	14	Very High
GC	UP	**ADD3**	Q9UEY8	hsa-miR-33a-5p	0.714639136946976	14	Very High
GC	UP	**ENC1**	O14682	hsa-miR-33a-5p	0.709386125143639	14	Very High
GC	UP	**KIAA1549L**	Q6ZVL6	hsa-miR-33a-5p	0.709117811116825	14	Very High
GC	UP	**HMGCR**	P04035	hsa-miR-33a-5p	0.694917209353761	14	Very High
GC	UP	**BCL11A**	Q9H165	hsa-miR-33a-5p	0.692895388441213	14	Very High
GC	UP	**FOXP1**	Q9H334	hsa-miR-33a-5p	0.692330467135263	14	Very High
GC	UP	**RPGRIP1L**	Q68CZ1	hsa-miR-33a-5p	0.691872258968482	14	Very High
GC	UP	**NFIX**	Q14938	hsa-miR-33a-5p	0.691439266213022	14	Very High
GC	UP	**SOX11**	P35716	hsa-miR-33a-5p	0.686841380406741	14	Very High
GC	UP	**AXL**	P30530	hsa-miR-33a-5p	0.686463302244065	14	Very High
GC	UP	**NPAT**	Q14207	hsa-miR-33a-5p	0.682597678139282	14	Very High
GC	UP	**ONECUT2**	O95948	hsa-miR-33a-5p	0.681323183600326	14	Very High
GC	UP	**PHF6**	Q8IWS0	hsa-miR-33a-5p	0.671919168054988	14	Very High
GC	UP	**ATXN7L1**	Q9ULK2	hsa-miR-33a-5p	0.667596481444746	14	Very High
GC	UP	**NPC1**	O15118	hsa-miR-33a-5p	0.809541927783163	13	Very High
GC	UP	**PIM1**	P11309	hsa-miR-33a-5p	0.797370894652071	13	Very High
GC	UP	**ESCO1**	Q5FWF5	hsa-miR-33a-5p	0.78725945980892	13	Very High
GC		**PALM2AKAP2**		hsa-miR-33a-5p	0.779372684467609	13	Very High
GC	UP	**GAS1**	P54826	hsa-miR-33a-5p	0.773026225549796	13	Very High
GC	UP	**GRIK5**	Q16478	hsa-miR-33a-5p	0.769054866842885	13	Very High
GC	UP	**ANO4**	Q32M45	hsa-miR-33a-5p	0.751271091279813	13	Very High
GC	UP	**WDR47**	O94967	hsa-miR-33a-5p	0.750525161761976	13	Very High
GC	UP	**MAP3K7**	O43318	hsa-miR-33a-5p	0.748308515495412	13	Very High
GC	UP	**NAV3**	Q8IVL0	hsa-miR-33a-5p	0.747228986486257	13	Very High
GC	UP	**SCN8A**	Q9UQD0	hsa-miR-33a-5p	0.746015317025356	13	Very High

GC	UP	IRS2	Q9Y4H2	hsa-miR-33a-5p	0.735757728679167	13	Very High
GC	UP	ZC3H12C	Q9C0D7	hsa-miR-33a-5p	0.734189912120867	13	Very High
GC	UP	LIMCH1	Q9UPQ0	hsa-miR-33a-5p	0.729105057194617	13	Very High
GC	UP	ST18	O60284	hsa-miR-33a-5p	0.723998118175107	13	Very High
GC	UP	SLITRK3	O94933	hsa-miR-33a-5p	0.721826728969059	13	Very High
GC	UP	ANKRD44	Q8N8A2	hsa-miR-33a-5p	0.720389172378059	13	Very High
GC	UP	PPM1B	O75688	hsa-miR-33a-5p	0.717292344204047	13	Very High
GC	UP	FBXO34	Q9NWN3	hsa-miR-33a-5p	0.717260026538486	13	Very High
GC	UP	CPSF2	Q9P2I0	hsa-miR-33a-5p	0.714453022955389	13	Very High
GC	UP	TENT5C	Q5VWP2	hsa-miR-33a-5p	0.714229130014276	13	Very High
GC	UP	SYT11	Q9BT88	hsa-miR-33a-5p	0.713516474385073	13	Very High
GC	UP	CNTN4	Q8IWV2	hsa-miR-33a-5p	0.712492926474351	13	Very High
GC	UP	USP46	P62068	hsa-miR-33a-5p	0.706386035339836	13	Very High
GC	UP	AFF3	P51826	hsa-miR-33a-5p	0.705696670277606	13	Very High
GC	UP	MEIS2	O14770	hsa-miR-33a-5p	0.705370249451901	13	Very High
GC	UP	ABHD13	Q7L211	hsa-miR-33a-5p	0.699144018423861	13	Very High
GC	UP	PNMA1	Q8ND90	hsa-miR-33a-5p	0.697777832760743	13	Very High
GC	UP	SLC16A1	P53985	hsa-miR-33a-5p	0.69454061832786	13	Very High
GC	UP	CCNYL1	Q8N7R7	hsa-miR-33a-5p	0.69400979026391	13	Very High
GC	UP	PCNX1	Q96RV3	hsa-miR-33a-5p	0.689926841258464	13	Very High
GC	UP	EEA1	Q15075	hsa-miR-33a-5p	0.689368782236545	13	Very High
GC	UP	FAM126B	Q8IXS8	hsa-miR-33a-5p	0.689314008982685	13	Very High
GC	UP	NIPAL4	Q0D2K0	hsa-miR-33a-5p	0.687017578532227	13	Very High
GC	UP	PDE7A	Q13946	hsa-miR-33a-5p	0.686609610132376	13	Very High
GC	UP	MRPS25	P82663	hsa-miR-33a-5p	0.68445453104227	13	Very High
GC	UP	TPM3	P06753	hsa-miR-33a-5p	0.681745226605537	13	Very High
GC	UP	RIMBP2	O15034	hsa-miR-33a-5p	0.680530249635495	13	Very High
GC	UP	GOPC	Q9HD26	hsa-miR-33a-5p	0.67926375494347	13	Very High
GC	UP	RLIM	Q9NVW2	hsa-miR-33a-5p	0.678238324617551	13	Very High
GC	UP	HOMER1	Q86YM7	hsa-miR-33a-5p	0.677430547208841	13	Very High
GC	UP	HOXD10	P28358	hsa-miR-33a-5p	0.676840508463508	13	Very High
GC	UP	RNF180	Q86T96	hsa-miR-33a-5p	0.676179060204681	13	Very High
GC	UP	PHACTR2	O75167	hsa-miR-33a-5p	0.674280314721226	13	Very High
GC	UP	SGCD	Q92629	hsa-miR-33a-5p	0.672527323023496	13	Very High
GC	UP	DSC2	Q02487	hsa-miR-33a-5p	0.672313098046524	13	Very High
GC	UP	BMP3	P12645	hsa-miR-33a-5p	0.661773725037827	13	Very High
GC	UP	DENND1B	Q6P3S1	hsa-miR-33a-5p	0.657463395847631	13	Very High
GC	UP	CSMD1	Q96PZ7	hsa-miR-33a-5p	0.775925543636374	12	Very High

GC	UP	**ZMIZ1**	Q9ULJ6	hsa-miR-33a-5p	0.743876605812557	12	Very High
GC	UP	**PPP3CB**	P16298	hsa-miR-33a-5p	0.720671371731464	12	Very High
GC	UP	**SEC24C**	P53992	hsa-miR-33a-5p	0.708682744468415	12	Very High
GC	UP	**KPNA3**	O00505	hsa-miR-33a-5p	0.706043332080738	12	Very High
GC	UP	**VCAN**	P13611	hsa-miR-33a-5p	0.705231650591481	12	Very High
GC	UP	**CELF2**	O95319	hsa-miR-33a-5p	0.705140694622369	12	Very High
GC	UP	**USP6**	P35125	hsa-miR-33a-5p	0.702689227479954	12	Very High
GC	UP	**ZNF148**	Q9UQR1	hsa-miR-33a-5p	0.698814640242705	12	Very High
GC	UP	**RASA1**	P20936	hsa-miR-33a-5p	0.698006668556923	12	Very High
GC	UP	**NUFIP2**	Q7Z417	hsa-miR-33a-5p	0.695437427958795	12	Very High
GC	UP	**CDK8**	P49336	hsa-miR-33a-5p	0.694856292481227	12	Very High
GC	UP	**HTATSF1**	O43719	hsa-miR-33a-5p	0.691746407716907	12	Very High
GC	UP	**CHD1**	O14646	hsa-miR-33a-5p	0.687037960060703	12	Very High
GC	UP	**SOCS5**	O75159	hsa-miR-33a-5p	0.686981031447256	12	Very High
GC	UP	**NR4A2**	P43354	hsa-miR-33a-5p	0.68442718859425	12	Very High
GC	UP	**DDX3X**	O00571	hsa-miR-33a-5p	0.679219747817588	12	Very High
GC	UP	**RGMA**	Q96B86	hsa-miR-33a-5p	0.677420285700025	12	Very High
GC	UP	**SNX9**	Q9Y5X1	hsa-miR-33a-5p	0.676689589677662	12	Very High
GC	UP	**RORA**	P35398	hsa-miR-33a-5p	0.676346677436103	12	Very High
GC	UP	**ADO**	Q96SZ5	hsa-miR-33a-5p	0.676188401598755	12	Very High
GC	UP	**ZFHX4**	Q86UP3	hsa-miR-33a-5p	0.6761554879157	12	Very High
GC	UP	**PTGFRN**	Q9P2B2	hsa-miR-33a-5p	0.674458386515443	12	Very High
GC	UP	**ST3GAL2**	Q16842	hsa-miR-33a-5p	0.672932573917488	12	Very High
GC	UP	**ADAMTS3**	O15072	hsa-miR-33a-5p	0.665873237538468	12	Very High
GC	UP	**CHN2**	P52757	hsa-miR-33a-5p	0.662291395945119	12	Very High
GC	UP	**ZFP36L1**	Q07352	hsa-miR-33a-5p	0.661533233161121	12	Very High
GC	UP	**QKI**	Q96PU8	hsa-miR-33a-5p	0.660719926751745	12	Very High
GC	UP	**DPP10**	Q8N608	hsa-miR-33a-5p	0.659353664692274	12	Very High
GC	UP	**C18orf25**	Q96B23	hsa-miR-33a-5p	0.659069266551081	12	Very High
GC	UP	**RAB5A**	P20339	hsa-miR-33a-5p	0.658760370257518	12	Very High
GC	UP	**CEP350**	Q5VT06	hsa-miR-33a-5p	0.658682918857139	12	Very High
GC	UP	**LDHA**	P00338	hsa-miR-33a-5p	0.70521354516442	11	Very High
GC	UP	**PFN2**	P35080	hsa-miR-33a-5p	0.695201974068175	11	Very High
GC	UP	**CALML4**	Q96GE6	hsa-miR-33a-5p	0.694137007365908	11	Very High
GC	UP	**S1PR1**	P21453	hsa-miR-33a-5p	0.684275574686861	11	Very High
GC	UP	**TCF7L2**	Q9NQB0	hsa-miR-33a-5p	0.682520371746094	11	Very High
GC	UP	**SLC26A7**	Q8TE54	hsa-miR-33a-5p	0.680721376514604	11	Very High
GC	UP	**TMEM86A**	Q8N2M4	hsa-miR-33a-5p	0.678413934193939	11	Very High

GC	UP	**CPEB2**	Q7Z5Q1	hsa-miR-33a-5p	0.677564494864291	11	Very High
GC	UP	**TSHZ1**	Q6ZSZ6	hsa-miR-33a-5p	0.675968323993514	11	Very High
GC	UP	**UNC79**	Q9P2D8	hsa-miR-33a-5p	0.670753764451196	11	Very High
GC	UP	**CREBZF**	Q9NS37	hsa-miR-33a-5p	0.659205376500232	11	Very High
GC	UP	**SIRT6**	Q8N6T7	hsa-miR-33a-5p	0.717607561942434	10	Very High
GC	UP	**AKAP2**	Q9Y2D5	hsa-miR-33a-5p	0.693632356347283	10	Very High
GC	UP	**CUL5**	Q93034	hsa-miR-33a-5p	0.669462087203863	10	Very High
GC	UP	**MAP3K1**	Q13233	hsa-miR-33a-5p	0.665587236598833	10	Very High
GC	UP	**GPR88**	Q9GZN0	hsa-miR-33a-5p	0.663941921424727	10	Very High
GC	UP	**NSF**	P46459	hsa-miR-33a-5p	0.663508600352738	10	Very High
